# Simultaneous Broadband Suppression of Homonuclear and Heteronuclear Couplings in ^1^H NMR Spectroscopy

**DOI:** 10.1002/cphc.202200495

**Published:** 2022-11-14

**Authors:** Coral Mycroft, Mathias Nilsson, Gareth A. Morris, Laura Castañar

**Affiliations:** ^1^ Department of Chemistry The University of Manchester Oxford Road Manchester M13 9PL United Kingdom

**Keywords:** analytical methods, fluorine, NMR spectroscopy, structure elucidation, phosphorus

## Abstract

The ^1^H NMR analysis of species containing NMR‐active heteronuclei can be difficult due to signal overlap caused by the combined effects of homonuclear and heteronuclear scalar (*J*) couplings. Here, a general pure shift method is presented for obtaining ultra‐high resolution ^1^H NMR spectra where spectral overlap is drastically reduced by suppressing both homonuclear and heteronuclear *J*‐couplings, giving one single signal per ^1^H chemical environment. Its usefulness is demonstrated in the analysis of fluorine‐ and phosphorus‐containing compounds of pharmaceutical and biochemical interest.

## Graphical abstract

**Ultra‐high resolution** ^**1**^ **H NMR** spectra are obtained by simultaneous broadband suppression of both homonuclear and heteronuclear scalar couplings, giving a fully pure shift ^1^H NMR spectrum containing chemical shift information only. The analysis of a range of fluorine‐ and phosphorus‐containing systems highlights how this method can be used to aid the analysis of even the most complex systems.

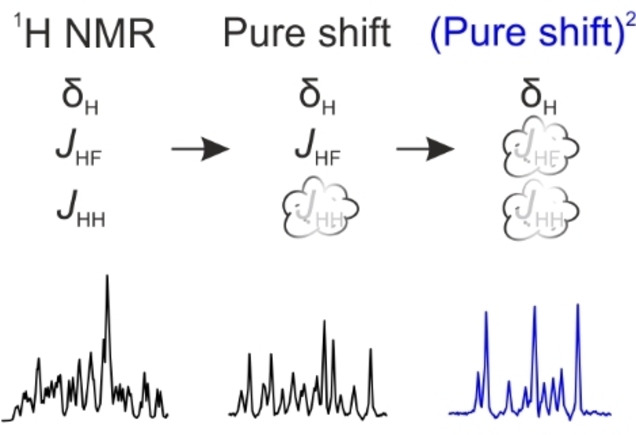

## Introduction

^1^H NMR is one of the most commonly used spectroscopic techniques as it provides valuable information on chemical structure and conformation. Because of the narrow spectral range of ^1^H, significant peak overlap due to multiplet structure caused by scalar (*J*) couplings is common, reducing spectral resolution and hindering the ability to extract useful information. Pure shift NMR techniques[^1^, ^2^, ^3^, ^4^] greatly improve spectral resolution by suppressing the effects of homonuclear *J*_HH_ couplings in ^1^H NMR spectra. These methods can lead to a single signal for each chemical site, but only if no other abundant NMR‐active nuclei such as ^19^F and ^31^P are present in the spin system. NMR measurements on compounds containing these particular nuclei are increasingly widespread due to their importance in pharmaceuticals[^5^, ^6^, ^7^] and biochemistry.[^8^, ^9^] ^19^F and ^31^P have a natural abundance of 100 % and are spin‐1/2 , so in ^1^H NMR their heteronuclear couplings cause multiplet structure in just the same way as homonuclear couplings. In a conventional pure shift NMR spectrum, heteronuclear couplings are still present, complicating analysis. Here we present a general method that simultaneously suppresses both homonuclear couplings and all heteronuclear couplings with a given isotope, giving a fully pure shift NMR spectrum.

## Results and Discussion

**Figure 1:**
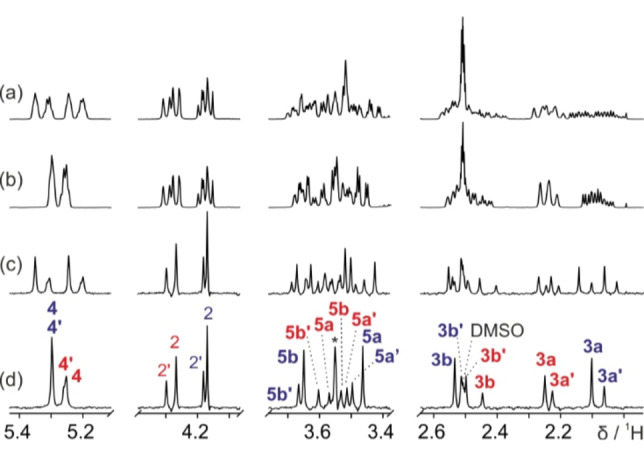
500 MHz ^1^H NMR spectra of a 90 mM solution of (4*R*)‐*N*‐Boc‐L‐fluoroproline and 87 mM of (4*S*)‐*N*‐Boc‐L‐fluoroproline (Scheme 1) in DMSO‐*d*_6_, showing only the regions of interest. (a) ^1^H NMR, (b) ^1^H{^19^F} NMR, (c) PSYCHE pure shift, and (d) PSYCHE pure shift measured using the new method for simultaneous homonuclear and heteronuclear decoupling. The asterisk indicates a strong coupling artefact. Structural assignments are shown in (d), where peaks with suppressed *J*_HF_ couplings are highlighted in bold. Further experimental details and full spectra are given in the Supporting Information.

**Scheme 1:**
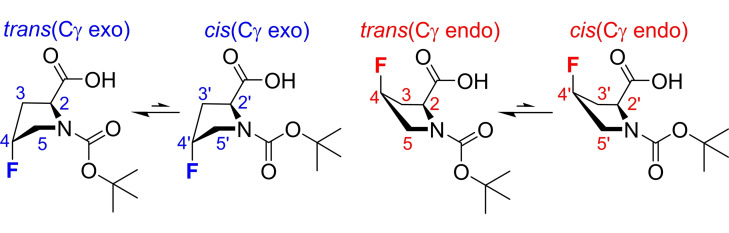
(left) 2 major rotamers of (4*R*)‐*N*‐Boc‐L‐fluoroproline and (right) 2 major rotamers of (4*S*)‐*N*‐Boc‐L‐fluoroproline. Trans/cis refers to the amide rotation and Cγ exo/endo refers to the pyrrolidine ring pucker conformation.

An example of the sort of signal overlap caused by simultaneous homonuclear and heteronuclear couplings is shown in the ^1^H NMR spectrum (Figure 1a) of a diastereomeric mixture of *N*‐Boc‐L‐4‐fluoroprolines (Scheme 1). Fluoroprolines have a wide range of applications in protein and peptide conformational studies.[^8^, ^10^, ^11^] They typically exist in multiple conformations, leading to very complex ^1^H NMR spectra. Couplings to ^19^F can easily be suppressed in conventional ^1^H NMR spectra by heteronuclear decoupling during signal acquisition, leaving only homonuclear couplings.[^12^, ^13^, ^14^, ^15^] However, despite the reduction in peak complexity, severe signal overlap is still observed (Figure 1b). Structural assignment of individual proton environments is near impossible, particularly in the region between 2.0 and 3.7 ppm. An alternative approach to reducing spectral complexity is to use a pure shift method to remove the effects of homonuclear coupling, leaving only those of heteronuclear coupling,[^1^, ^2^, ^3^, ^4^] as seen in Figure 1c. For simple molecules, pure shift methods can be very useful for measuring heteronuclear coupling constants.[^16^, ^17^, ^18^, ^19^] However, in more complex systems, even after suppressing all homonuclear couplings, heteronuclear multiplicity remains a problem for spectral analysis. For example, in the fluoroproline mixture studied, only with the aid of the fully decoupled pure shift NMR spectrum (Figure 1d), measured using the new method presented here, is the full anatomy of the spectrum exposed.

At first sight, the solution seems obvious: suppress heteronuclear couplings in a pure shift NMR spectrum by applying broadband decoupling to ^19^F during signal acquisition, just as in the conventional ^1^H{^19^F} NMR spectrum. Unfortunately, it is not sufficient only to irradiate ^19^F during signal acquisition since heteronuclear couplings evolve throughout the pure shift pulse sequence (see Supporting Information, Figure S9). Application of broadband heteronuclear decoupling throughout the pulse sequence would collapse the multiplicity but can be impractical due to the use of field gradient pulses, as their application greatly increases the range of Larmor frequencies. A real‐time pure shift method published by Lokesh *et al*. for selective measurement of individual ^1^H‐^19^F couplings^[20]^ could in principle allow fully ^19^F decoupled pure shift ^1^H NMR spectra to be obtained. However, this application has not yet been demonstrated and the method would not be suitable for large *J*_HF_ values and/or wide ^19^F chemical shift ranges. There is also an existing pure shift experiment that routinely incorporates heteronuclear decoupling, the BIRD method,^[21]^ but the decoupling mechanism is specific to the BIRD pulse sequence element, has limited heteronuclear bandwidth, and is not generally applicable.

**Figure 2:**
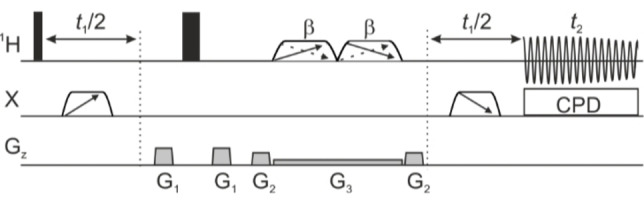
Generally‐applicable pulse sequence for heteronuclear decoupled 1D pure shift NMR. Narrow and wide filled rectangles denote hard 90° and 180° radiofrequency pulses, respectively. Trapezoids with cross‐diagonal arrows denote low‐power saltire^[23]^ chirp pulses of nominal flip angle β (∼20°). Trapezoids on the heteronuclear (X) channel denote frequency‐swept adiabatic 180° pulses. When *t*_1_=0, these frequency‐swept pulses are not applied. Broadband adiabatic decoupling (CPD) is applied during acquisition. G_1_ and G_2_ represent pulsed field gradients for coherence transfer pathway selection, and G_3_ denotes a weak rectangular pulsed field gradient. Here, the PSYCHE pure shift version of the experiment is shown, but band‐selective and Zangger‐Sterk elements can be used in place of PSYCHE; these are coded as options in the pulse program code provided in the Supporting Information.

Here, we present a general pure shift NMR approach in which both homonuclear and heteronuclear *J*‐couplings are efficiently suppressed over a wide range of heteronuclear coupling constants and chemical shifts, by refocussing *J*_HX_ prior to decoupling. The new method provides a true pure shift ^1^H NMR spectrum, in which peak positions are determined solely by chemical shifts. As shown in Figure 1d, the new method yields a significant improvement in resolution when applied to the fluoroproline mixture. In the region 3.4–3.7 ppm, the complex multiplet patterns caused by *J*_HF_ couplings are greatly simplified, allowing each chemical shift to be distinguished, and subsequently assigned with the aid of conventional 1D and 2D experiments. The proposed fully decoupled interferogram pure shift method is shown in Figure 2. The sequence shown uses the PSYCHE approach,[^22^, ^23^] but the same logic can be applied using Zangger‐Sterk,^[24]^ band‐selective[^25^, ^26^, ^27^] or BIRD elements.^[28]^ An adiabatic heteronuclear 180° pulse is applied midway through each *t*_1_/2 evolution period, refocusing the evolution of the heteronuclear couplings. The use of adiabatic pulses, counter‐sweeping to refocus chemical shift evolution during the pulses, ensures efficient inversion over the wide chemical shift ranges typically encountered for nuclei such as ^19^F and ^31^P. Just as in the conventional ^1^H{^19^F} NMR experiment, broadband adiabatic decoupling is applied during acquisition. This approach ensures that the effects of all heteronuclear couplings, whether large or small, are suppressed in a fully pure shift ^1^H NMR spectrum. In pure shift experiments, the price for signal simplification is a reduction in sensitivity, typically to about 5–20 % of that of the conventional ^1^H NMR spectrum. The new method does not incur any extra cost in experiment time compared with the conventional interferogram pure shift experiment, and increases the signal‐to‐noise ratio of the decoupled signals by at least a factor of two.

As previously mentioned, an alternative and simpler strategy, used by Lokesh *et al*.,^[20]^ is to treat the heteronuclear couplings in the same way as homonuclear couplings, simply adding a hard X 180° pulse simultaneously with the hard ^1^H 180° pulse in a standard pure shift sequence (see Supporting Information, Section 1b). However, this approach only gives good results when the magnitudes of heteronuclear couplings are no greater than those of homonuclear couplings (see Supporting Information, Figure S6) and the heteronucleus chemical shift range is relatively narrow, although it does away entirely with the need for broadband X irradiation, minimising sample heating. With large *J*_HF_ values, such as those in the fluoroproline compounds, using this approach results in large artefacts and imperfectly decoupled signals (see Supporting Information, Figure S7).

**Scheme 2:**
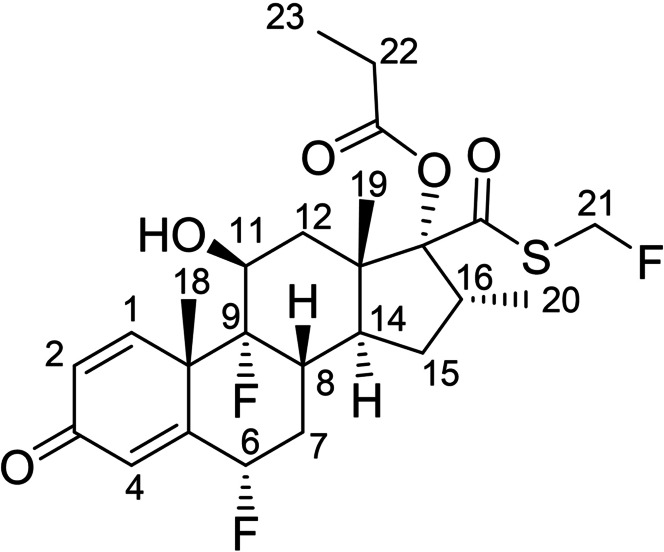
Fluticasone propionate.

**Figure 3:**
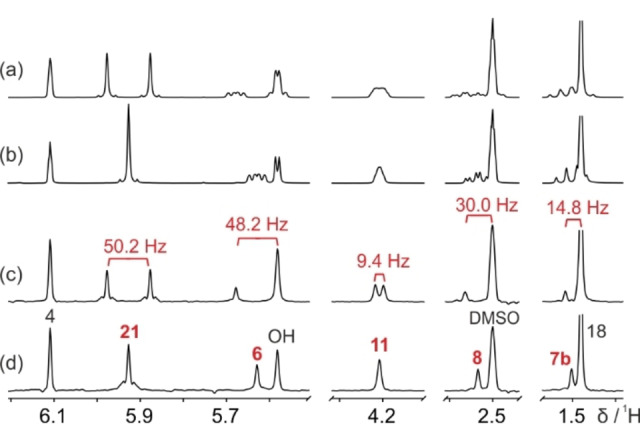
500 MHz ^1^H NMR spectra of a 22 mM solution of fluticasone propionate (Scheme 2) in DMSO‐*d*_6_, showing only the regions of interest. (a) ^1^H NMR, (b) ^1^H{^19^F} NMR, (c) pure shift PSYCHE, and (d) fully decoupled pure shift PSYCHE. *J*_HF_ couplings are shown in (c). Structure assignment is shown in (d), where peaks with suppressed *J*_HF_ coupling are highlighted in red. Further experimental details and full spectra are given in the Supporting Information.

The general applicability of the new method is demonstrated in the analysis of fluticasone propionate (Scheme 2), a trifluoroglucocorticoid used in the treatment of asthma and allergic rhinitis.[^29^, ^30^] Here, peak overlap is less severe than in the previous example, but the presence of three different fluorine chemical environments spanning over 30 ppm poses a different challenge. The conventional ^1^H NMR spectrum (Figure 3a) shows poor spectral resolution in several regions due to the combination of homonuclear and heteronuclear couplings, e. g. for protons 7b (1.51 ppm), 8 (2.54 ppm) and 6 (5.64 ppm). Resolution is improved when either heteronuclear or homonuclear decoupling is applied (Figures 3b and 3c, respectively), but neither method fully resolves every chemical site. Only when the new method is applied, removing the effects of both homonuclear and heteronuclear couplings, is a true pure shift ^1^H NMR spectrum obtained in which each distinct chemical site is represented by a single peak (Figure 3d). With a 30 ppm ^19^F chemical shift range, the adiabatic pulses in the new method ensure effective inversion of all ^19^F resonances (see Supporting Information, Figure S8).

**Scheme 3:**
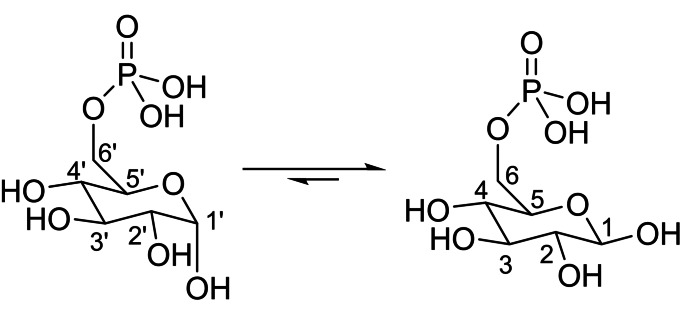
(left) α‐D‐glucose‐6‐phosphate and (right) β‐D‐glucose‐6‐phosphate.

**Figure 4:**
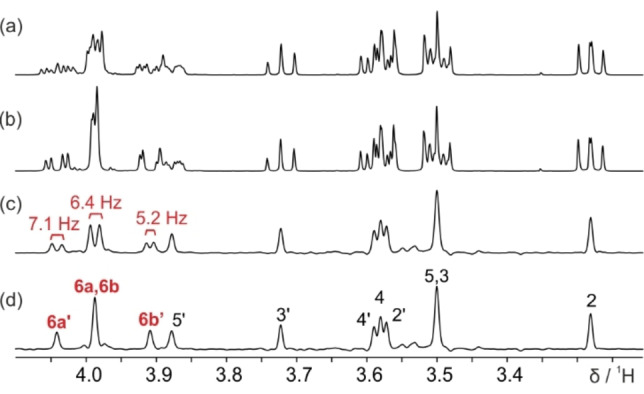
500 MHz ^1^H NMR spectra of a 30 mM solution of D‐glucose‐6‐phosphate in D_2_O, in which signals are seen from both α and β anomers (Scheme 3). (a) ^1^H NMR, (b) ^1^H{^31^P} NMR, (c) pure shift PSYCHE, and (d) fully decoupled pure shift PSYCHE. *J*_HP_ couplings are shown in Figure 4c. Structure assignment is shown in (d), where peaks with suppressed *J*_HP_ coupling values are highlighted in red. Further experimental details are given in the Supporting Information.

This new pure shift method is a general tool that can easily be applied to decouple other nuclei with large chemical shift ranges and/or wide ranges of *J*_HX_ coupling values. Possible other examples include carbon‐13, silicon‐29 and phosphorus‐31. Here, its applicability to ^31^P is demonstrated on the key metabolite D‐glucose‐6‐phosphate (Scheme 3).^[31]^ With heteronuclear couplings *J*_HP_ comparable in magnitude to *J*_HH_ couplings, and with most of the protons resonating within 1 ppm, spectral interpretation is difficult even for this relatively simple molecule. In water, the equilibrium between the α and β anomeric forms^[32]^ complicates the ^1^H NMR spectrum further (Figure 4a). Phosphorus decoupling (Figure 4b) gives only a small improvement. The standard pure shift NMR spectrum (Figure 4c) drastically reduces signal overlap, allowing protons H4’, H4 and H2’ at 3.6 ppm to be distinguished. All signals are fully decoupled when the new method is applied (Figure 4d).

## Conclusion

Here, we have presented a general pure shift experiment that allows the effects of homonuclear and heteronuclear scalar couplings to be simultaneously suppressed for a wide range of heteronuclear chemical shifts and couplings. This general tool is suitable for decoupling high‐abundance nuclei, including fluorine‐19 and phosphorus‐31, over the full range of chemical shifts and heteronuclear coupling constants. Its usefulness has been demonstrated in the analysis of fluorine‐ and phosphorus‐containing pharmaceutical and biochemical molecules, yielding a significant increase in resolution when compared to conventional pure shift methods, without increasing experiment time. Such experiments can be used to aid complex sample or mixture analysis, be easily applied to other NMR‐active nuclides to suppress *J*_HX_ couplings in pure shift ^1^H NMR spectra, and have potential applications throughout chemistry, biochemistry, and chemical biology.

## Experimental Section

### Sample Preparation

All compounds used were commercially available from Sigma‐Aldrich and were used without further purification. Fluoroproline sample contained a mixture of 15.7 mg of (4*R*)‐*N*‐Boc‐L‐fluoroproline and 15.3 mg of (4*S*)‐*N*‐Boc‐L‐fluoroproline dissolved in 750 μL of DMSO‐*d*_6_. Fluticasone sample contained 8.1 mg of fluticasone propionate dissolved in 750 μL of DMSO‐*d*_6_. Glucose mixture contained 6.4 mg of D‐glucose‐6‐phosphate sodium salt dissolved in 750 μL of D_2_O. The sample was left overnight before analysis to ensure that equilibrium between α and β anomeric forms was reached.

### Data Acquisition

All experimental spectra were recorded at 298 K on a Bruker Avance NEO 500 MHz NMR spectrometer with a 5 mm TBI probe equipped with a z‐gradient coil with a maximum nominal gradient strength of 67 G cm^−1^. Conventional ^1^H NMR experiments were recorded with 5 kHz spectral width and 16k complex points. The duration of the hard 90° pulse was set to 12.80, 13.00 and 13.93 μs for the fluoroproline, fluticasone and glucose samples, respectively. For heteronuclear decoupling during acquisition, adiabatic decoupling sequence ‘p5 m4sp180.2’ was applied, with a WURST pulse with 80 % smoothing, a Q factor of 3, and a duration of 3.82, 3.94 and 6 ms for the fluoroproline, fluticasone and glucose samples, respectively. 1D pure shift spectra were recorded with 5 kHz spectral width, 64, 20 and 20 *t*_1_ increments and 12800, 4000 and 4000 complex points for the fluoroproline, fluticasone and glucose samples, respectively. A chunk duration of 20 ms was used in all pure shift experiments. Adiabatic WURST inversion pulses with 20 % smoothing were applied during the *t*_1_ incremented delays, with a duration of 3 ms and a Q factor of 3. Pure shift PSYCHE data was acquired using a double saltire pulse with a flip angle of 20°, 20°, 15° and a total duration of 200, 60 and 200 ms for the fluoroproline, fluticasone and glucose samples, respectively. G_1_ and G_2_ are half‐sine shaped gradient pulses with amplitudes of 52.9 and 31.5 G cm^−1^, respectively, and a duration of 1 ms each. G_3_ is a rectangular gradient pulse, aligned with the midpoint of the double saltire pulse, and has an amplitude of 0.67 G cm^−1^. Further experimental details and pulse program codes for Bruker spectrometers are given in the Supporting Information.

### Data Processing

All data was processed with zero‐filling, Gaussian line broadening, Fourier transformation, and phase and baseline correction using the TOPSPIN program (Bruker Biospin). Pure shift data was processed using the reconstruction macro *pshift4f*.

All experimental data, pulse program codes, macros and experimental parameters are freely available at https://doi.org/10.48420/19583323.

## Conflict of interest

The authors declare no conflict of interest.

1

## Supporting information

As a service to our authors and readers, this journal provides supporting information supplied by the authors. Such materials are peer reviewed and may be re‐organized for online delivery, but are not copy‐edited or typeset. Technical support issues arising from supporting information (other than missing files) should be addressed to the authors.

Supporting Information

## Data Availability

The data that support the findings of this study are openly available in Figshare at https://doi.org/10.48420/19583323, reference number 19583323.
