# Supplementary material for: Simultaneous Broadband Suppression of Homonuclear and Heteronuclear Couplings in 1H NMR Spectroscopy
Source: Chemphyschem. 2022 Nov 14;23(24):e202200495. doi: 10.1002/cphc.202200495 (PMC10099583; doi:10.1002/cphc.202200495)
Supplement: Supplementary file 1 — Supporting Information [file CPHC-23-0-s001.pdf]

ChemPhysChem

Supporting Information

Simultaneous Broadband Suppression of Homonuclear and Heteronuclear Couplings in ^{1}H NMR Spectroscopy

Coral Mycroft, Mathias Nilsson, Gareth A. Morris, and Laura Castañar*

Table of Contents

1. Pulse sequence descriptions……………………………………………………… 2
   a. Generally-applicable pulse sequence…………………………………… 2
   b. 'Small J and δ' pulse sequence………………………………………….. 4
2. Shaped pulses……………………………………………………………………… 6
   a. Using Wavemaker to generate shaped pulses………………………… 6
   b. Creating saltire pulses for PSYCHE pure shift…………………………. 7
3. Recommended protocol…………………………………………………………… 8
4. Experimental data………………………………………………………………….. 9
   a. (4*R*)-N-Boc-L-fluoroproline and (4*S*)-N-Boc-L-fluoroproline sample…. 9
   b. Fluticasone propionate sample…………………………………………... 11
   c. D-glucose-6-phosphate disodium salt sample………………………….. 13
5. Comparisons 15
   a. Comparison between the 'generally-applicable' and the 'small J and δ' pulse sequences………………………………………………………… 15
   b. The effect of applying heteronuclear decoupling during acquisition only 19
6. Pulse program codes for Bruker spectrometers………………………………… 20
   a. Generally-applicable pulse program suitable for a TBI probe………… 20
   b. 'Small J and δ' pulse program suitable for a TBI probe………………… 24
7. References…………………………………………………………………………… 27

1. Pulse sequence descriptions

The pulse sequence presented in the main paper is referred to below as the 'generally-applicable pulse sequence' as it can be used for any range of chemical shift and any value of J_{HX} coupling. An alternative sequence is described in Section 1b as the 'small J and δ' sequence. This sequence is only recommended for small J_{HX} values and a small heteronuclear chemical shift (δ) range. As a rule of thumb, if the chemical shift range is larger than 1/(8 p1) in Hz, where p1 is the X nucleus hard 90° pulse duration, the generally-applicable pulse sequence is recommended.

The pulse sequences below are described for Bruker probes (in this case a TBI probe) using standard transmitter routing. Pulse program codes suitable for a QNP probe are available from the authors on request.

a. Generally-applicable pulse sequence

The detailed generally-applicable pulse sequence is shown in Figure S1 (pulse program code can be found in Section 6a). Narrow and wide filled rectangles represent hard 90° and 180° pulses, respectively. The durations of the hard 90° pulses (p1) for each sample are given in Section 4. The two trapezoids with diagonal arrows in the ^{n}X (^{19}F or ^{31}P) channel represent counter-sweeping WURST adiabatic inversion pulses (with durations p15 and p19 and shape names spnam15 and spnam19, respectively). The use of adiabatic pulses with opposite sweep directions (spnam15 and spnam19) is necessary to ensure that chemical shift evolution during these pulses is refocused. In the first increment (when t_1= 0), these pulses are not applied. The pulses were designed using *Wavemaker* (see Section 2a) to invert all heteronuclear resonances over the required chemical shift range. Trapezoids with cross-diagonal arrows in the ^{1}H channel represent saltire pulses with duration p40. These are low-power chirp pulses with a net off-resonance flip angle β of approximately 20° that sweep frequency simultaneously in opposite directions. Two consecutive saltire pulses are known as a double saltire pulse. A description of how to design a double saltire pulse is given in Section 2b. Band-selective,[1–3] Zangger-Sterk[4] and PSYCHE[5,6] active spin refocusing elements are available within the pulse program (see Section 6). The shaped pulses required for these elements can be designed using *Wavemaker* as described in Section 2a.

The pulsed field gradient G_{10} is applied simultaneously with the double saltire pulse, with duration p10 and rectangular shape (gpnam10 = RECT.1), and it is followed by a short recovery delay (d17) of 200 µs. Pulsed field gradients G_1 and G_2 are used to select the desired coherence transfer pathway. They have amplitudes of 79% (52.9 G cm^{-1}) and 47% (31.5 G cm^{-1}) respectively, a duration (p16) of 1 ms, and a half-sine shape (gpnam1 = gpnam2 = SINE.100). G_1 and G_2 are followed by a recovery delay (d16) of 1 ms.

The relaxation delay (d_1) is recommended to be at least $2T_1$, where T_1 is the longitudinal spin-relaxation time constant of the relevant ^{1}H signals. The d0 delays are incremented by in steps of $1/(2SW_1)$, half the chunk duration. The delay τ_B is required to compensate for the number of data points (cnst4) that need to be discarded after the start of data acquisition to avoid data corruption by the effect of the digital filtration. The number of dropped points (cnst4) used was 4. The delay τ_A ensures that J_{HH} couplings are refocused at the midpoint of each chunk of data acquisition. The chunk duration ($1/SW_1$) is dependent on the magnitude of J_{HH} couplings ($1/SW_1 << 1/J_{HH}$) and is typically set to 20 ms. The delay Δ is equal to (d0 – p15)/2. The adiabatic composite pulse decoupling (CPD) sequence "p5m4sp180.2" was applied in the X channel during acquisition, using adiabatic WURST-80 pulses which were designed using *Wavemaker*. The recommended minimum phase cycle is 2 steps; the full phase cycle is given in Table S1.

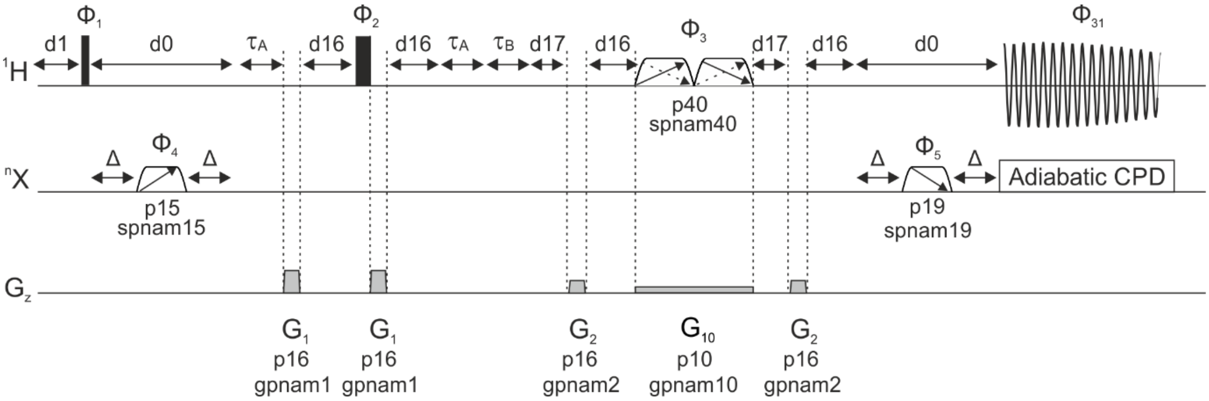

Figure S1: Detailed pulse sequence for Bruker implementation of the generally-applicable fully decoupled pure shift experiment proposed in the main paper. Pulse program code can be found in Section 6a. The definition of each parameter can be found at the end of the pulse program, along with recommended standard values. Adiabatic composite pulse decoupling is applied in the X channel during acquisition. Here, the PSYCHE pure shift version of the experiment is shown, but band-selective and Zangger-Sterk elements can be used in place of PSYCHE.

Table S1: Phase cycle for the pulse sequence described in Figure S1.

**Φ_1**	x_4, y_4
**Φ_2**	x_8, $-x_8$
**Φ_3**	x, y, -x, -y
**Φ_4**	x
**Φ_5**	x
**Φ_{31}**	$(x, -x)_2$, $(y, -y)_2$

b. ‘Small J and δ’ pulse sequence

The pulse sequence described below (Figure S2) is an alternative pulse sequence to the generally-applicable method previously described. Here, a hard X 180° pulse is applied simultaneously with the hard ^{1}H 180° pulse, so J_{HX} couplings evolve in the same way as J_{HH} couplings. No decoupling is applied during acquisition, and no adiabatic X pulses are applied during the d0 evolution periods. This pulse sequence is easier to set up than the generally-applicable pulse sequence described above (Figure S1) as default parameters for the hard X 180° pulse can be used, however careful pulse calibration is recommended. It is essential that the heteronuclear resonances experience a good 180° X pulse, therefore, the X pulse must be applied close to resonance. In the case of multiple heteronuclear signals with very different Larmor frequencies, the resultant spectrum is likely to exhibit significant artefacts due to imperfect suppression of the effects of J_{HX} coupling.

This method generates chunking artefacts at the same positions as conventional interferogram[4,7] pure shift experiments (±n SW_1 Hz), with the presence of heteronuclear couplings increasing the intensity of the chunking artefacts observed in the pure shift NMR spectrum. To ensure that these chunking artefacts do not interfere with spectral analysis, this pulse sequence is only recommended for use when J_{HX} values are comparable to or less than J_{HH} values.

The detailed ‘small J and δ’ pulse sequence is shown in Figure S2 (pulse program code can be found in Section 6b). Narrow and wide filled rectangles represent hard 90° and 180° pulses, respectively. The durations of the hard 90° pulses (p1) for each sample are given in Section 4. Trapezoids with cross-diagonal arrows in the ^{1}H channel represent saltire pulses (with duration p40). These are low-power chirp pulses with a net off-resonance flip angle β of approximately 20° that sweep frequency simultaneously in opposite directions. Two consecutive saltire pulses are known as a double saltire pulse. A description of how to design a double saltire pulse is given in Section 2b. Band-selective,[1–3] Zangger-Sterk[4] and PSYCHE[5,6] active spin refocusing elements are available within the pulse program (see Section 6b). The selective pulses required for these elements can be designed using *Wavemaker* as described in Section 2a.

The pulsed field gradient G_{10} is applied simultaneously with the double saltire pulse, with duration p10 and rectangular shape (gpnam10 = RECT.1), and it is followed by a short recovery delay (d17) of 200 µs. Pulsed field gradients G_1 and G_2 are used to select the desired coherence transfer pathway. They have amplitudes of 79% (52.9 G cm^{-1}) and 47% (31.5 G cm^{-1}) respectively, a duration (p16) of 1 ms, and a half-sine shape (gpnam1 = gpnam2 = SINE.100). G_1 and G_2 are followed by a recovery delay (d16) of 1 ms.

The relaxation delay (d_1) is recommended to be at least $2T_1$, where T_1 is the longitudinal spin-relaxation time constant of the relevant ^{1}H signals. The d0 delays are incremented by in steps of $1/(2SW_1)$, half the chunk duration. The delay τ_B is required to compensate for the number of data points (cnst4) that need to be discarded after the start of data acquisition to avoid data corruption by the effect of the digital filtration. The number of dropped points (cnst4) used was 4. The delay τ_A ensures that J_{HH} couplings are refocused at the midpoint of each chunk of data acquisition. The chunk duration ($1/SW_1$) is dependent on the magnitude of J_{HH} couplings ($1/SW_1 << 1/J_{HH}$) and is typically set to 20 ms. The recommended minimum phase cycle is 2 steps; the full phase cycle is given in Table S2.

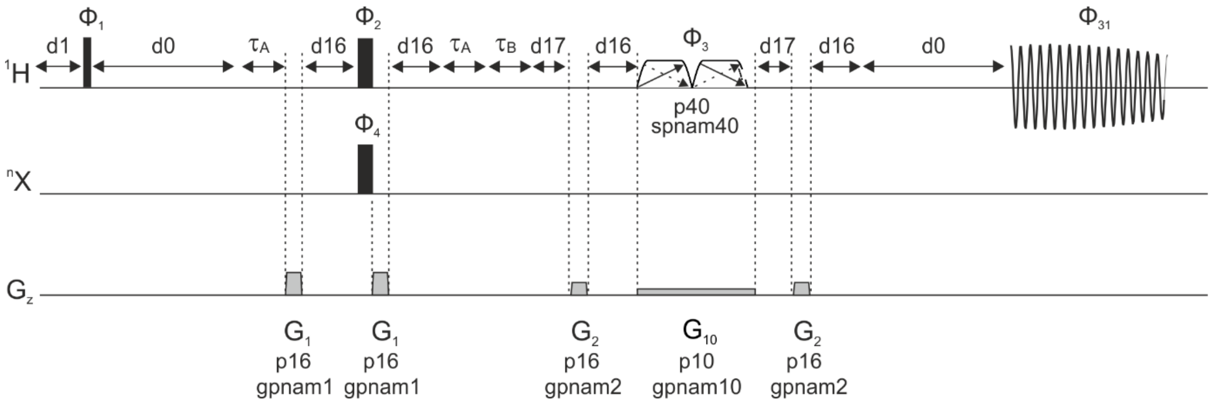

Figure S2: Detailed pulse sequence for Bruker implementation of the 'small J and δ' fully decoupled pure shift experiment. Pulse program code can be found in Section 6b. The definition of each parameter can be found at the end of the pulse program, along with recommended standard values. This pulse sequence is only recommended when the magnitude of J_{HX} couplings is comparable to the magnitude of J_{HH} couplings and over a small heteronuclear chemical shift range. Here, the PSYCHE pure shift version of the experiment is shown, but band-selective and Zangger-Sterk elements can be used in place of PSYCHE.

Table S2: Phase cycle for the pulse sequence described in Figure S2.

**Φ_1**	x_4, y_4
**Φ_2**	x_8, $-x_8$
**Φ_3**	x, y, -x, -y
**Φ_4**	x
**Φ_{31}**	$(x, -x)_2$, $(y, -y)_2$

2. Shaped pulses

a. Using *Wavemaker* to generate shaped pulses

Wavemaker is a pulse shaping function within Topspin® that designs shaped pulses without the need for extensive manual set-up and allows for easy adjustment of pulse parameters. *Wavemaker* reads the relevant lines within the pulse program, and from the parameters specified designs the shaped pulses required. In the generally-applicable pulse sequence, there are 4 shaped pulses designed by *Wavemaker*:

- Adiabatic WURST-20 and WURST-80 pulses are used for X (f2 channel), for the inversion pulses (sp15 and sp19) and for the adiabatic decoupling sequence (sp31), respectively.
- If the user uses the band-selective or Zangger-Sterk pure shift option, an rSNOB shape (sp12) will be used for the refocusing pulse. (Note that if PSYCHE is used, *Wavemaker* still designs an rSNOB pulse but it is not used).

The relevant lines in the pulse program are shown below:

```
;sp15:wvm:X1_wurst-20:f2 wurst-20(cnst5 kHz, cnst3 ms; Q=cnst7, L2H)
;sp19:wvm:X2_wurst-20:f2 wurst-20(cnst5 kHz, cnst3 ms; Q=cnst7, H2L)
;sp31(p63,pl12):wvm:HXdec:f2 wurst-80(cnst5 kHz, cnst10 ms; Q=cnst8, L2H) ss=1.0 us;

;sp12:wvm:H_rsnob:f1 rsnob(cnst12 Hz, cnst11 ppm; PA=0.5; NPOINTS=1000) ss=1.0 us;
```

Wavemaker reads the parameters ‘cnst5’ (for X) and ‘cnst12’ (for ^{1}H) to define the effective bandwidths in kHz and Hz respectively. For X, the effective bandwidth must cover the full heteronuclear chemical shift range. For ^{1}H, the effective bandwidth must cover only one frequency or a range of frequencies within which spins are not coupled to each other. ‘cnst3’ and ‘cnst10’ (for X) specify the durations of the adiabatic pulses. ‘Q’ is the adiabaticity factor of each pulse. For ^{1}H, ‘cnst11’ specifies the chemical shift at which the rSNOB pulse is to be applied.

To generate the shaped pulses, the command “*wvm –a”* must be used following any changes to the parameters.

Further information can be found in the *Wavemaker* manual in the Bruker User Library. Alternatively, each pulse can be set up manually.

b. <u>Creating saltire pulses for PSYCHE pure shift</u>

As the active spin refocusing element used in the PSYCHE approach may be unfamiliar, a detailed description on how to design the double saltire pulse in Topspin® software version 4.0.7 is given. Details of how to create the double saltire pulse in Topspin® software version 2.1 can be found in the original pure shift PSYCHE publication.[1,2]

In Topspin®, open the 'Shape Tool' using the command 'stdisp' and select an 'adiabatic smoothed chirp' shaped pulse. Under 'General parameters', set 'size [pt]' to 10000 points and pulse length to 15 ms. Under 'Waveform parameters', set the sweep width to 10 kHz (or at least double the desired spectral window), 20% to be smoothed, and low-to-high (LTH) field. Save the shape ('Chirp1') and create an identical smoothed chirp pulse ('Chirp2') with high-to-low (HTL) field (by deselecting the LTH field).

Open 'Chirp1' then open 'Chirp2' to combine them into a single shape. Align the HTL pulse to the end of the LTH pulse, by setting the 'offset [pt]' of the HTL pulse to 10000 points. Under 'Combined Shape', set the 'scaling method' to 100% and save as 'AddShape1'. Align the LTH pulse to the end of the HTL pulse by setting the 'offset [pt]' of the LTH pulse to 10000 points and the 'offset [pt]' of the HTL pulse to 0 points. Under 'Combined Shape', set the 'scaling method' to 100% and save as 'AddShape2'.

Open 'AddShape1' then open 'AddShape2' to combine them into a single shape. Under combined shape, scale to 100%. Save this pulse as 'PSYCHE_saltire_30m_10kHz' and set as 'spnam10'. Define the PSYCHE element duration (p10) as 30 ms, the flip angle (cnst20) as 10-25° and the bandwidth (cnst21) as 10000 Hz (or the defined sweep width).

The parameters provided will be suitable for acquiring PSYCHE pure shift data for most cases. However, if further optimisation is needed, changes to the pulse duration and bandwidth of the double saltire pulse can be made using the Shapetool function. The flip angle β can be modified simply by changing the RF amplitude of the double saltire pulse without altering its shape.

3. Recommended protocol

When setting up the generally-applicable pulse sequence for the first time in the NMR spectrometer, it is recommended to follow the protocol below, which assumes that the f2 channel is set to ^{19}F. For ^{31}P, follow the same protocol but with ^{31}P instead of ^{19}F. When setting up the 'small J and δ' pulse sequence, steps 3 and 5 - 8 are not required.

1. Acquire the ^{1}H NMR spectrum and calibrate the 90° hard pulse.
2. Acquire the ^{19}F NMR spectrum and calibrate the 90° hard pulse if possible, moving the transmitter to the same offset as a peak of your choice. Once calibrated, move the transmitter to the middle of the range required.
3. Set up a $^{1}H\{^{19}F\}$ NMR experiment with adiabatic decoupling, using CPD program 'p5m4sp180.2' or a suitable alternative. Create an adiabatic WURST pulse shape (duration p63, shape sp31) with 80% smoothing with the following parameters:
    a. An effective bandwidth larger than the ^{19}F chemical shift range.
    b. A pulse duration of less than $1/(5J_{HF})$, to minimise J_{HF} evolution during acquisition.

   A $^{1}H\{^{19}F\}$ NMR pulse sequence that uses the CPD program 'p5m4sp180.2' and WURST pulses with 80% smoothing is available at DOI: 10.48420/19583323.

4. Compare the ^{19}F coupled and decoupled spectra to try to determine the magnitude of the largest J_{HF} value in Hz. If this cannot be determined, an estimate of e.g. 50 Hz can be used.
5. Acquire a conventional pure shift spectrum using an appropriate ASR element (band-selective, Zangger-Sterk or PSYCHE). Optimise the chunk duration, number of chunks and ASR element for the sample to be analysed.
6. The parameters previously extracted from steps 2-4 (J_{HF}, ^{19}F chemical shift range) can then be used to set up the adiabatic inversion pulses within the fully decoupled pure shift ^{1}H NMR experiment.
7. Heteronuclear decoupling can cause an increase in temperature, particularly with pulses optimised for a large bandwidth and/or large J_{HF} values. If possible, increase the adiabatic pulse duration or reduce the Q factor to minimise fluctuations in temperature. Note that the duration of the ^{19}F inversion WURST pulses (p15 and p19) must not exceed half the chunk duration.
8. The duration of the adiabatic decoupling sequence (cnst10) should not be longer than $1/(5J_{HF})$ (cnst9) to minimise J_{HF} evolution during acquisition. If possible, a shorter time is recommended.
9. Finally, run the new experiment to obtain a ^{1}H NMR spectrum in which the effects of both J_{HF} and J_{HH} couplings have been suppressed.

4. Experimental data

All raw experimental data for this paper are freely available for download from https://doi.org/10.48420/19583323.

All NMR spectra shown in the main manuscript were recorded at 298 K on a Bruker Avance NEO 500 MHz NMR spectrometer with a 5 mm TBI probe equipped with a z-gradient coil with a maximum nominal gradient strength of 67 G cm^{-1}. All fully decoupled pure shift NMR spectra in the main manuscript were recorded using the generally-applicable sequence. For full assignment, conventional 1D and 2D NMR spectra (i.e., ^{1}H, $^{19}F/^{31}P$, ^{1}H -^{1}H COSY, ^{1}H-^{13}C HSQC and ^{1}H-^{13}C HMBC) were also acquired (data not shown). The pulse programs given below are for a TBI probe (codes suitable for a QNP probe are available from the authors on request).

a. <u>(4*R*)-*N*-Boc-L-fluoroproline and (4*S*)-*N*-Boc-L-fluoroproline sample</u>

15.7 mg of commercial (4*R*)-*N*-Boc-L-fluoroproline (or *N*-Boc-*trans*-4-fluoro-L-proline) (Sigma-Aldrich) and 15.3 mg of commercial (4*S*)-*N*-Boc-L-fluoroproline (or *N*-Boc-*cis*-4-fluoro-L-proline) (Sigma-Aldrich) were dissolved in 750 µL of DMSO-d_6.

For ^{1}H NMR experiments, the ^{1}H spectral window was set to 5000 Hz (10 ppm), the carrier frequency to 1600.42 Hz (3.2 ppm), and the duration of the hard 90° pulse to 12.80 µs. 1D ^{1}H NMR data were acquired with 1 transient, a time domain data length (TD) of 16384, and with an experiment time of 8 s. Prior to Fourier transformation of all 1D ^{1}H NMR data, zero-filling to 65536 complex points was applied, and Gaussian weighting with LB of -0.01 Hz and GB of 0.00163 was used.

For ^{19}F experiments, the ^{19}F spectral window were set to 5000 Hz (10.6 ppm), the carrier frequency to -81823.31 Hz (-173.9 ppm), and the duration of the hard 90° pulse to 26.35 µs. 1D ^{19}F NMR data were acquired with 1 transient, a TD of 16384, and with an experiment time of 8 s. $\{^{1}H\}^{19}F$ NMR spectra were acquired using the CPD sequence WALTZ16[8] with a pulse duration of 80 µs and power level of 0.23535 W. Prior to Fourier transformation of 1D ^{19}F NMR data, zero-filling to 65536 was applied, and Gaussian weighting with LB of -0.01 Hz and GB of 0.00643 was used.

Pure shift ^{1}H NMR spectra were recorded with 5 kHz spectral width, 64 t_1 increments, a chunk duration of 20 ms (SW_1 = 50 Hz), 12800 complex points, and 8 transients in an experiment time of 43 min and 33 s. Pure shift PSYCHE data were acquired using a double saltire pulse with a total duration of 200 ms, a flip angle of 20° and a gradient of 1% (0.67 G cm^{-1}). (A long double saltire pulse duration of 200 ms was required to minimise strong coupling artefacts).

Prior to Fourier transformation of pure shift ^{1}H NMR data, zero-filling to 16384 was applied, and Gaussian weighting with LB of -0.01 Hz and GB of 0.000163 was used.

For ^{19}F decoupling in 1D ^{1}H NMR and pure shift ^{1}H NMR, the adiabatic decoupling sequence 'p5m4sp180.2' was used, with a WURST pulse with 80% smoothing, a duration of 3.82 ms and a Q factor of 3. Adiabatic WURST inversion pulses with 20% smoothing were applied during the t_1 incremented delays d0, with a duration of 3 ms and a Q factor of 3.

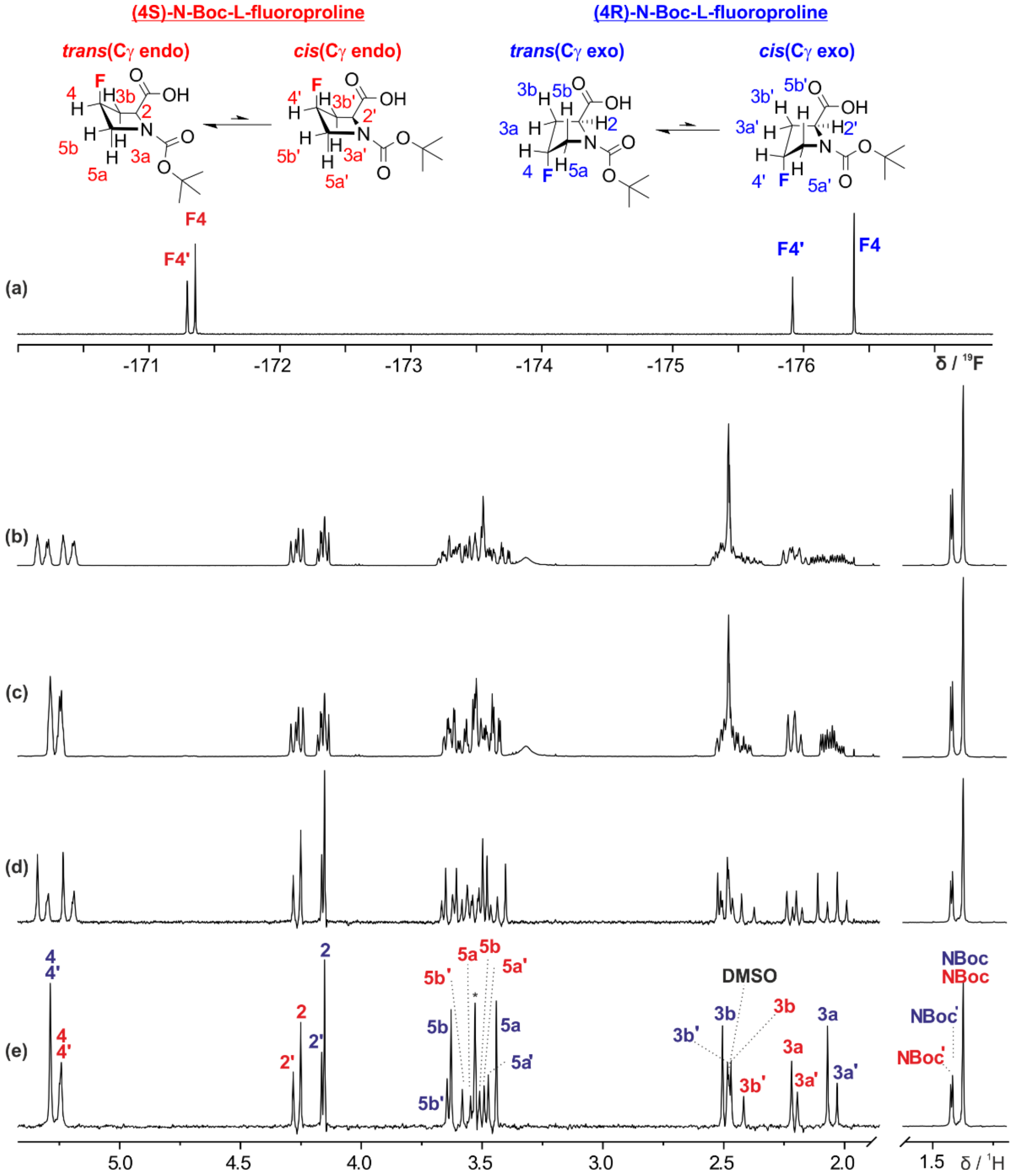

Figure S3: (Top) Chemical structures and (bottom) 500 MHz NMR spectra of 90 mM (4*R*)-*N*-Boc-L-fluoroproline and 87 mM (4*S*)-*N*-Boc-L-fluoroproline in DMSO-d_6. (a) ^{19}F{^{1}H} NMR, (b) conventional ^{1}H NMR, (c) ^{1}H{^{19}F} NMR, (d) pure shift PSYCHE and (e) fully decoupled pure shift PSYCHE NMR spectra. Structural assignment is shown in spectrum (e). The Boc group region was scaled down by a factor of 4 compared to the rest of the spectrum because of the difference in signal intensity. The asterisk indicates a strong coupling artefact.

b. Fluticasone propionate sample

8.1 mg of commercial fluticasone propionate (Sigma-Aldrich) was dissolved in 750 µL of DMSO-d_6.

For ^{1}H NMR experiments, the ^{1}H spectral window were set to 5000 Hz (10 ppm), the carrier frequency to 2000 Hz (4 ppm), and the duration of the hard 90° pulse to 13.0 µs. 1D ^{1}H NMR data were acquired with 4 transients, a TD of 16384, and with an experiment time of 46 s. Prior to Fourier transformation of 1D ^{1}H NMR spectra, zero-filling to 65536 was applied, and Gaussian weighting with LB of -0.01 Hz and GB of 0.00163 was used.

For ^{19}F experiments, the ^{19}F spectral window were set to 30120 Hz (64 ppm), the carrier frequency to -83615 Hz (-177.68 ppm), and the duration of the hard 90° pulse to 26.2 µs. 1D ^{19}F NMR data were acquired with 16 transients, a TD of 32768, in an experiment time of 1 min 45 s. {^{1}H}^{19}F NMR spectra were acquired using the CPD sequence WALTZ16[8] with a pulse duration of 80 µs and power level of 0.23535 W. Prior to Fourier transformation of all 1D ^{19}F NMR data, zero-filling to 131072 was applied, and Gaussian weighting with LB of -0.01 Hz and GB of 0.00213 was used.

Pure shift ^{1}H NMR spectra were recorded with 5 kHz spectral width, 20 t_1 increments, a chunk duration of 20 ms (SW_1 = 50 Hz), 4000 complex points and 4 transients in an experiment time of 8 min 53 s. Pure shift PSYCHE data were acquired using a double saltire pulse with a total duration of 60 ms, a flip angle of 20° and a gradient of 1% (0.67 G cm^{-1}). Prior to Fourier transformation of pure shift ^{1}H NMR data, zero-filling to 16k was applied, and Gaussian weighting with LB of -0.01 Hz and GB of 0.00163 were applied.

For ^{19}F decoupling in 1D ^{1}H NMR and pure shift ^{1}H NMR, adiabatic decoupling sequence ‘p5m4sp180.2’ was used, with an WURST pulse with 80% smoothing, a duration of 3.94 ms and a Q factor of 3. Adiabatic WURST inversion pulses with 20% smoothing were applied during the t_1 incremented delays, with a duration of 3 ms and a Q factor of 3.

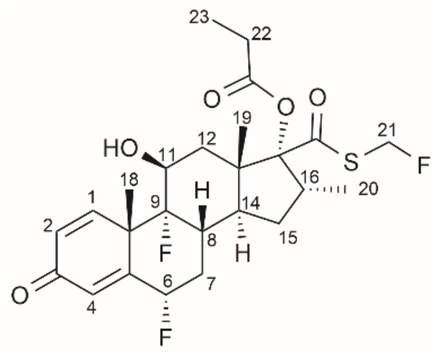

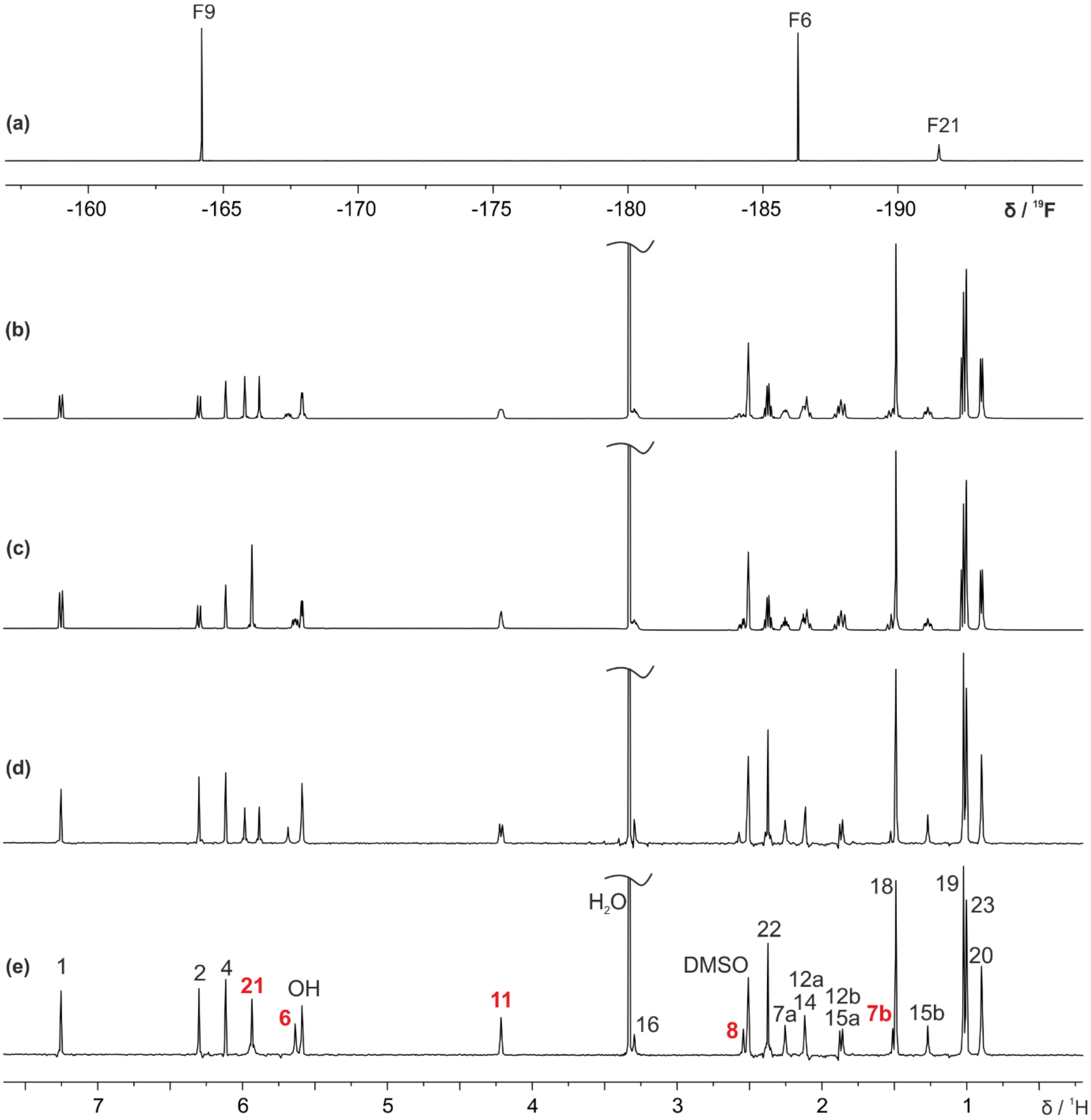

Figure S4: (Top) Chemical structure and (bottom) 500 MHz NMR spectra of 22 mM fluticasone propionate in DMSO-d_6. (a) $^{19}F\{^{1}H\}$ NMR, (b) conventional ^{1}H NMR, (c) $^{1}H\{^{19}F\}$ NMR, (d) pure shift PSYCHE and (e) fully decoupled pure shift PSYCHE NMR spectra. Structural assignment is shown in spectrum (e), where proton environments highlighted in red have a J_{HF} coupling present in spectra (b) and (d).

c. D-glucose-6-phosphate disodium salt sample

6.4 mg of commercial D-glucose-6-phosphate sodium salt (Sigma-Aldrich) was dissolved in 750 µL of D_2O. The sample was left overnight before analysis to ensure that equilibrium between α and β anomeric forms was reached.

For ^{1}H NMR experiments, the ^{1}H spectral window were set to 5000 Hz (10 ppm), the carrier frequency to 2050.53 Hz (4.1 ppm), and the duration of the hard 90° pulse to 13.925 µs. 1D ^{1}H NMR data were acquired with 16 transients and a TD of 16384 in an experiment time of 2 min 34 s. Prior to Fourier transformation of 1D ^{1}H NMR data, zero-filling to 65536 was applied, and Gaussian weighting with LB of -0.01 Hz and GB of 0.00643 was used.

For ^{31}P experiments, the ^{31}P spectral window were set to 2500 Hz (12.35 ppm), the carrier frequency to 911.05 Hz (4.5 ppm), and the duration of the hard 90° pulse was 27.875 µs. 1D ^{31}P NMR data were acquired with 128 transients, a TD of 8192, and with an experiment time of 20 min 37 s. {^{1}H}^{31}P NMR spectra were acquired using the CPD sequence WALTZ16[8] with a pulse duration of 80 µs and power level of 0.23535 W. Prior to Fourier transformation of 1D ^{31}P NMR spectra, zero-filling to 32768 was applied, and Gaussian weighting with LB of -0.01 Hz and GB of 0.00643 was used.

Pure shift ^{1}H NMR spectra were recorded with 5 kHz spectral width, 20 t_1 increments, a chunk duration of 20 ms (SW_1 = 50 Hz), 2048 complex points and 16 transients with an experiment time of 46 min and 22 s. Pure shift PSYCHE data were acquired using a double saltire pulse with a total duration of 200 ms, a flip angle of 15° and a gradient of 1% (0.67 G cm^{-1}). A long double saltire pulse duration of 200 ms was required to minimise strong coupling artefacts. Prior to Fourier transformation of pure shift ^{1}H NMR spectra, zero-filling to 16384 was applied, and Gaussian weighting with LB of -0.01 Hz and GB of 0.000163 was used.

For ^{31}P decoupling in 1D ^{1}H NMR and pure shift ^{1}H NMR, the adiabatic decoupling sequence 'p5m4sp180.2' was used, with a WURST pulse with 80% smoothing, a duration of 6 ms, and a Q factor of 3. Adiabatic WURST inversion pulses with 20% smoothing were applied during the t_1 incremented delays d0, with a duration of 3 ms and a Q factor of 3.

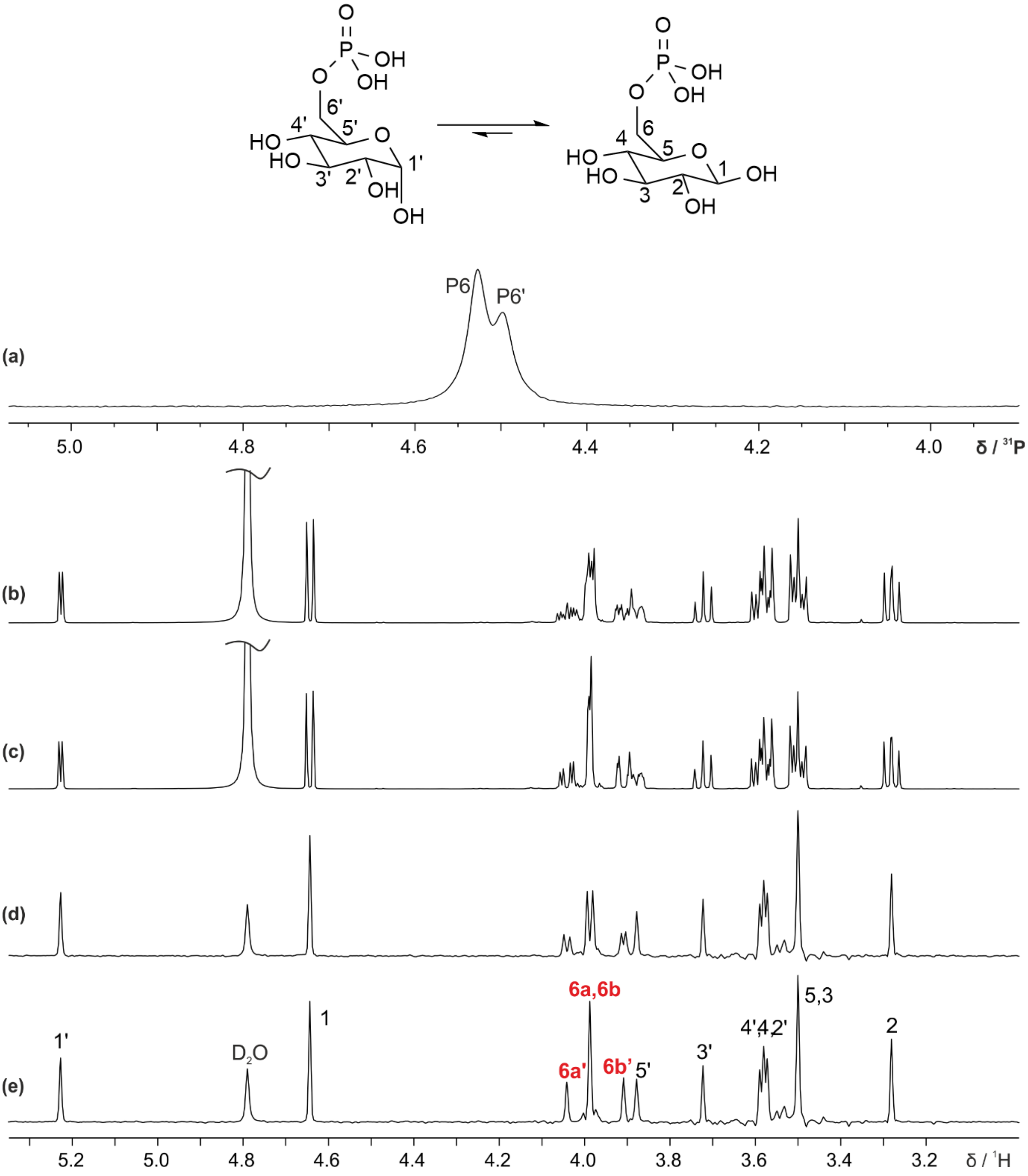

Figure S5: (Top) Chemical structures and (bottom) 500 MHz NMR spectra of 30 mM α-D-glucose-6-phosphate and β-D-glucose-6-phosphate in D_2O in an approximate ratio of 1:1.7. (a) $^{31}P\{^1H\}$ NMR, (b) conventional ^1H NMR, (c) $^1H\{^{31}P\}$ NMR, (d) pure shift PSYCHE and (e) fully decoupled pure shift PSYCHE NMR spectra. Structural assignment is shown in spectrum (e), where proton environments highlighted in red have a J_{HP} coupling present in spectra (b) and (d).

5. Comparisons

a. Comparison between the 'generally-applicable' and the 'small J and δ' pulse sequences

D-glucose-6-phosphate in D_2O exhibits J_{HP} couplings of 5-7 Hz (Table S3), comparable to the J_{HH} couplings, and a small ^{31}P chemical shift range of 0.2 ppm (see Figure S5a). In this case, the two pulse sequences (Figures S1 and S2) provide similar pure shift spectra (Figures S6b and c) in which the effects of both J_{HH} and J_{HP} coupling are suppressed, and little difference in spectral purity is observed. Due to the low signal-to-noise ratio, and the J_{HH} and J_{HP} couplings being much smaller than $1/SW_1$ ($1/SW_1 >> 1/J$), chunking artefacts are too small to be visible.

Table S3: Summary of J_{HP} couplings for D-glucose-6-phosphate in DMSO-d_6. J_{HP} couplings were obtained by comparing the PSYCHE and fully decoupled PSYCHE spectra in Figures S5b and c.

$^nJ_{H(X)P}$	$\|J\|$ / Hz
$^3J_{H6aP}$	6.4
$^3J_{H6bP}$	6.4
$^3J_{H6a'P'}$	7.1
$^3J_{H6b'P}$	5.2

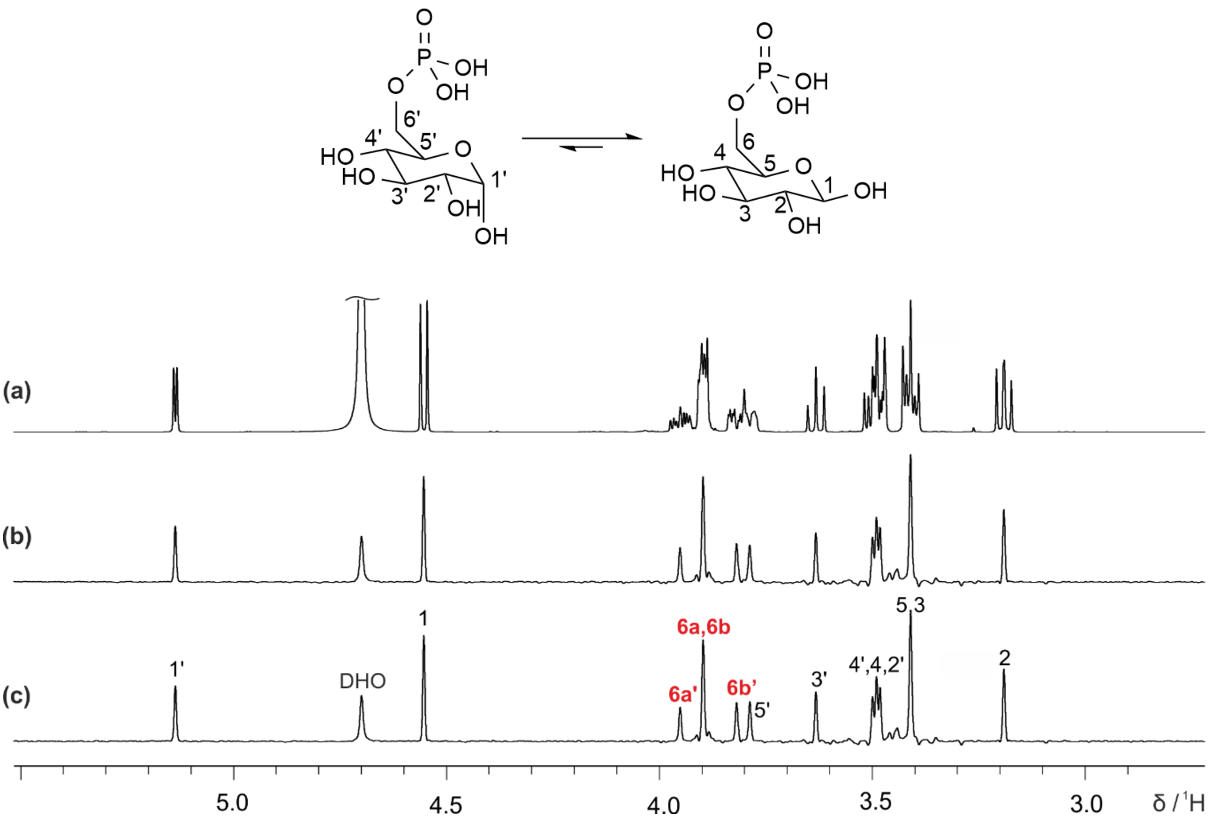

Figure S6: (Top) Chemical structures and (bottom) 500 MHz ^1H NMR spectra of 30 mM α- and β-D-glucose-6-phosphate in D_2O. (a) Conventional ^1H NMR, (b) fully decoupled pure shift PSYCHE using the generally-applicable sequence (Figure S1) and (c) fully decoupled pure shift PSYCHE using the 'small J and δ' sequence (Figure S2). Structural assignment is shown in spectrum (c).

To highlight the importance of using a suitable heteronuclear decoupled pure shift ^{1}H NMR pulse sequence, a comparison of the spectra provided by the two sequences for the *N*-Boc-4-fluoro-L-proline mixture is shown in Figure S7. With J_{HF} couplings of up to 54 Hz (Table S4), this sample highlights the problems with using the 'small J and δ' pulse sequence when J_{HF} >> J_{HH}.

In Figure 7b, where the generally-applicable pulse sequence (Figure S1) was used, the effects of all J_{HF} and J_{HH} couplings have clearly been suppressed in the pure shift spectrum. However in spectrum (c), where the 'small J and δ' pulse sequence (Figure S2) was used, the severe chunking artefacts caused by large J_{HF} couplings evolving during the data acquisition chunks make interpretation difficult, particularly in the region around 3.5 ppm corresponding to protons H5 and H5', with couplings of up to 54 Hz. For this mixture, the use of the generally-applicable sequence in Figure S7c is essential for obtaining a high-quality pure shift spectrum.

Table S4: Summary of couplings J_{HF} for (4*R*)-*N*-Boc-L-fluoroproline and (4*S*)-*N*-Boc-L-fluoroproline in DMSO-d_6. J_{HF} couplings were obtained by comparing the PSYCHE and fully decoupled PSYCHE in Figures S7b and c.

**Sample**	$^{n}J_{H(X)F}$	**|J | / Hz**
(4*S*)-*N*-Boc-L-fluoroproline	$^{3}J_{H3aF}$	19.5
	$^{3}J_{H3bF}$	43.2
	$^{2}J_{H4F}$	52.9
	$^{3}J_{H5aF}$	28.2*
	$^{3}J_{H5bF}$	36.4*
	$^{3}J_{H3a'F'}$	19.5
	$^{3}J_{H3b'F'}$	43.0
	$^{2}J_{H4'F'}$	53.1
	$^{3}J_{H5a'F'}$	28.0*
	$^{3}J_{H5b'F'}$	31.4*
(4*R*)-*N*-Boc-L-fluoroproline	$^{3}J_{H3aF}$	40.3
	$^{3}J_{H3bF}$	19.4
	$^{2}J_{H4F}$	52.9
	$^{3}J_{H5aF}$	37.9
	$^{3}J_{H5bF}$	22.0
	$^{3}J_{H3a'F'}$	39.7
	$^{3}J_{H3b'F'}$	20.9
	$^{2}J_{H4'F'}$	52.9
	$^{3}J_{H5a'F'}$	37.5
	$^{3}J_{H5b'F'}$	22.0

*Due to the strong J_{HH} coupling between 5a and 5b and between 5a' and 5b' in (4*S*)-N-Boc-L-proline, it is difficult to determine the J_{HF} coupling constants accurately; only approximate values are given.

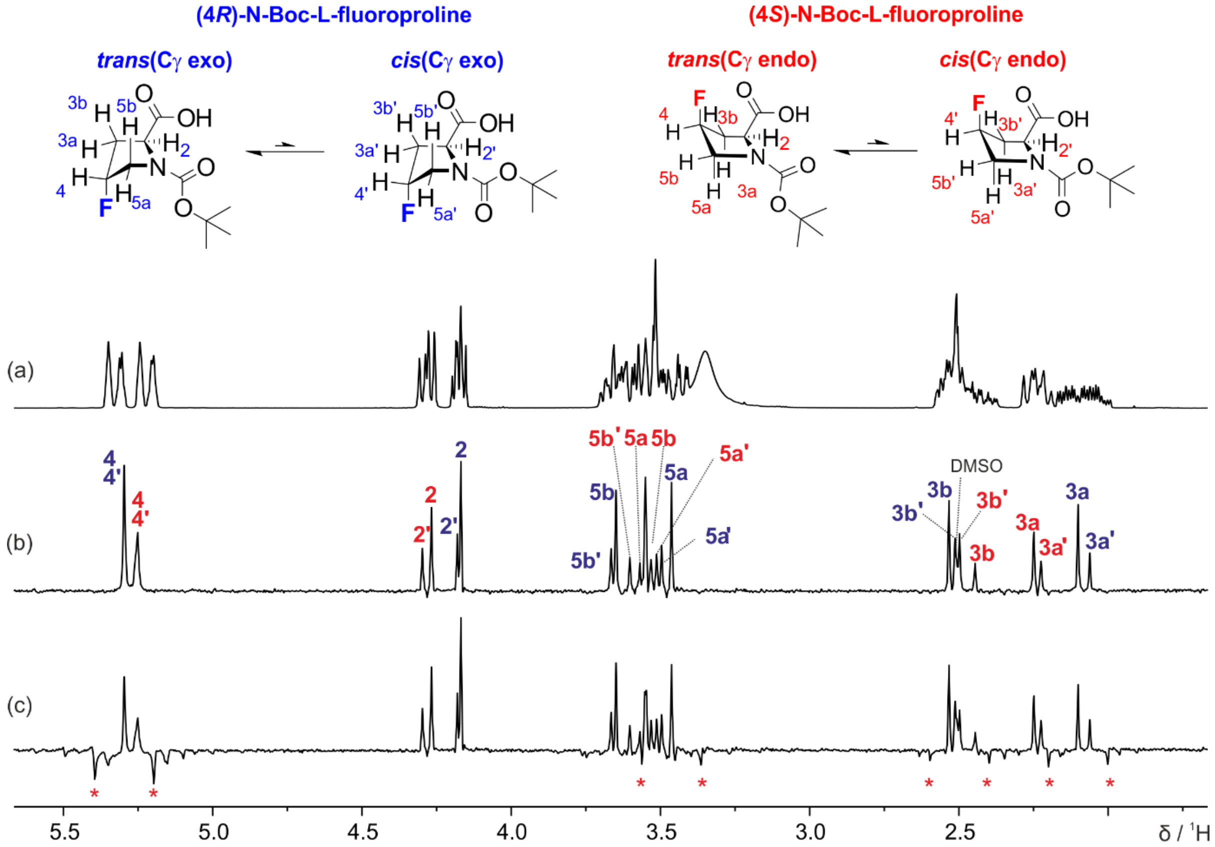

Figure S7: (Top) Chemical structures and (bottom) 500 MHz ^{1}H NMR spectra of a 90 mM solution of (left) (4*R*)-*N*-Boc-L-fluoroproline and 87 mM of (right) (4*S*)-*N*-Boc-L-fluoroproline in DMSO-d_6. (a) Conventional ^{1}H NMR, (b) fully decoupled pure shift PSYCHE using the generally-applicable sequence (Figure S1) and (c) fully decoupled PSYCHE using the 'small J and δ' sequence (Figure S2). Structural assignment is shown in spectrum (b). Significant chunking artefacts (highlight in spectrum (d) by red asterisks) are present at ±50n Hz from the parent peaks.

To further emphasise the importance of using the appropriate pulse sequence, a comparison between the spectra provided by the two methods for fluticasone propionate is shown in Figure S8. With three ^{19}F signals spanning over 30 ppm (-164.2, -186.3, and -191.5 ppm for F9, F6 and F21, respectively) and J_{HF} couplings of up to 51 Hz (Table S5), this sample highlights the issue of using the 'small J and δ' pulse sequence when multiple ^{19}F signals with very different shifts are present and when $J_{HF} >> J_{HH}$.

In Figure S8b, where the generally-applicable pulse sequence (Figure S1) was used, the effects of all J_{HF} and J_{HH} couplings have been suppressed with minimal artefacts in the pure shift spectrum. In spectrum (c), where the 'small J and δ' sequence (Figure S2) was used, J_{HF} coupling causes the signal corresponding to H21 to appear 'triplet-like' (left spectrum in Figure S8e) due to the poor off-resonance performance of the hard 180° ^{19}F pulse (14 ppm off resonance). The signal at 2.55 ppm, corresponding to H8, also appears to be 'triplet-like' (right spectrum in Figure S8e), but is difficult to see due to signal overlap with the DMSO peak at 2.5 ppm. In this sample, due to the large J_{HF} couplings and ^{19}F chemical shift range, the use of the generally-applicable sequence is again preferred.

Table S5: Summary of J_{HF} couplings for fluticasone propionate in DMSO-d_6. J_{HF} couplings were obtained from comparing PSYCHE and fully decoupled PSYCHE in Figure S4b and c.

$^nJ_{H(X)F}$	|J |/ Hz
$^2J_{H21F21}$	50.4
$^2J_{H6F6}$	49.1
$^3J_{H11F9}$	9.4
$^3J_{H8F9}$	29.8
$^3J_{H7bF6}$	14.5

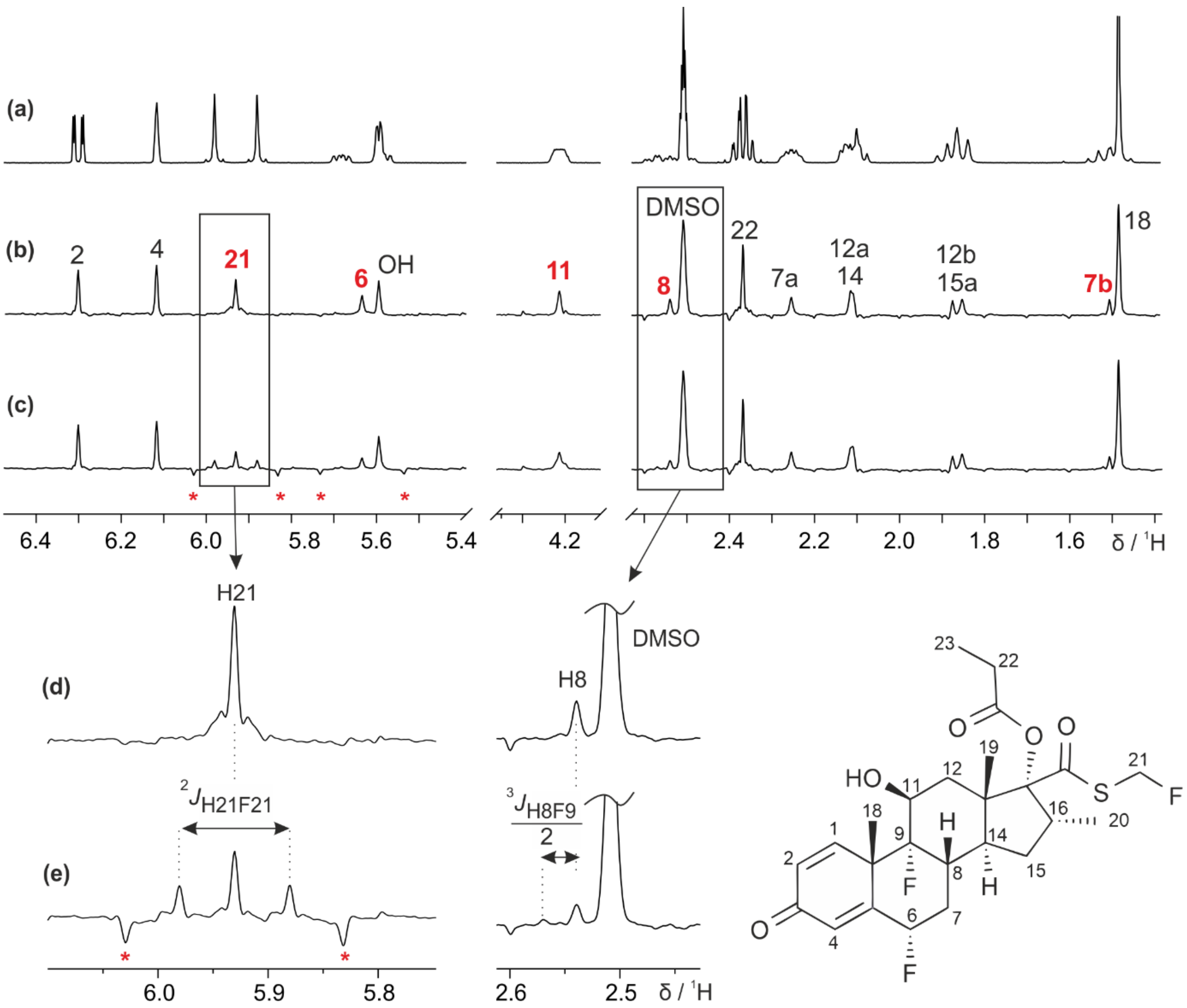

Figure S8: (a) 500 MHz conventional ^{1}H NMR, (b) fully decoupled pure shift PSYCHE using the generally-applicable pulse sequence and (c) fully decoupled PSYCHE using the 'small J and δ' sequence of 22 mM fluticasone propionate (bottom right) in DMSO-d_6. Spectra (d) and (e) are expansions of (b) and (c), focusing on signals coupled to F21 and F9, the signals furthest from resonance with the ^{19}F 180° pulse applied at -177.68 ppm. In spectra (c) and (e), chunking artefacts due to large J_{HF} couplings are highlighted by red asterisks, and unsuccessful suppression of the effects of J_{HF} coupling is highlighted at 5.9 ppm by a black box. In spectrum (e) at 2.55 ppm, the right-hand component of the H8 signal is underneath the DMSO peak. Structural assignment is shown in spectrum (b).

b. The effect of applying heteronuclear decoupling during acquisition only

As mentioned in the manuscript, to suppress the effect of heteronuclear scalar couplings in pure shift NMR spectra it is not sufficient simply to apply broadband heteronuclear decoupling during signal acquisition, since the signal evolution as a function of time t_1 that determines the form of the spectrum obtained will still include the effects of heteronuclear couplings. The result is a spectrum that (a) shows (phase-modulated) heteronuclear coupling structure, and (b) contains multiple strong sidebands (so-called chunking artefacts) that arise because the signal evolution in t_1 is inconsistent with that in t_2. Only when using the new method, in which heteronuclear couplings are refocused throughout the whole experiment, is a clean, fully decoupled, pure shift spectrum obtained (Figure S9d).

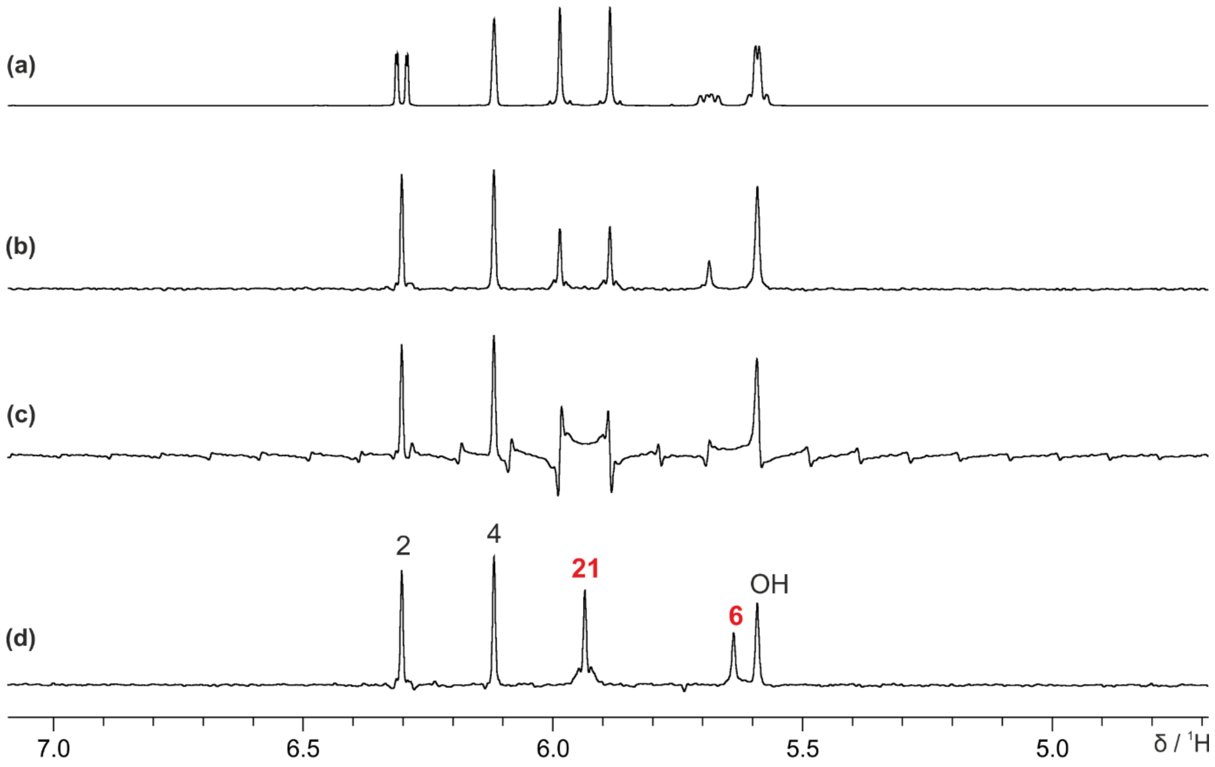

Figure S9: (a) 500 MHz conventional NMR of 22 mM fluticasone propionate in DMSO-d_6, showing only a region of interest: ^{1}H NMR, (b) pure shift PSYCHE, (c) pure shift PSYCHE with adiabatic heteronuclear decoupling applied during acquisition only and (d) fully decoupled PSYCHE using the 'generally-applicable' pulse sequence. Structural assignment is shown in spectrum (d), where signals with J_{HF} couplings are highlighted in red. In spectrum (c) the effect of heteronuclear couplings is still present and significant chunking artefacts are observed at 50 Hz intervals, corresponding to ±n SW_1 from parent peaks.

6. Pulse program codes for Bruker spectrometers

a. Generally-applicable pulse program suitable for a TBI probe

```
;X_dec_if_1d_ts4x_tbi
;
;Developed by:
;NMR Methodology Group
;University of Manchester
;
;Pure shift 1D experiment
;     with BB heteronuclear and homonuclear decoupling
;     with interferogram acquisition
;
;Data can be reconstructed using the "pshift4f" macro available at
https://www.nmr.chemistry.manchester.ac.uk/sites/default/files/pshift4f
;
;References:
;Heteronuclear decoupled: C. Mycroft, M. Nilsson, G. A. Morris, L. Castanar, Simultaneous broadband
suppression of homonuclear and heteronuclear couplings in 1H NMR spectroscopy (2022)
;Interferogram acquisition:  K. Zangger, H. Sterk, J. Magn. Reson., 124, 486-489 (1997)
;                            J. A. Aguilar, S. Faulkner, M. Nilsson, G. A. Morris, 49, 3901-3903 (2010)
;Band-selective: L. Castanar, P. Nolis, A. Virgili, T. Parella, Chem. Eur. J., 19, 17283-17286 (2013)
;                J. Ying, J. Roche, A. Bax, J. Magn. Reson., 241, 97-102 (2014)
;Zangger-Sterk:  K. Zangger, H. Sterk, J. Magn. Reson., 124, 486-489 (1997)
;PSYCHE: M. Foroozandeh, R. W. Admas, N. J. Meharry, D. Jeannerat, M. Nilsson, G. A. Morris, Angew. Chem.
Int. Ed., 53, 6990-6992 (2014)
;
;THE USE OF WAVEMAKER IS RECOMMENDED
;
;Pure shift active spin refocusing options
;      ZGOPTION= -DBS (Band-Selective)
;      ZGOPTION= -DZS (Zangger-Sterk)
;      ZGOPTION= -DdSALTIRE (PSYCHE) [used by default]
;
;Avance NEO Version
;Topspin.4x
;
;$CLASS=HighRes
;$DIM=2D
;$TYPE=
;$SUBTYPE=
;$COMMENT=
;

#include <Avance.incl>
#include <Delay.incl>
#include <Grad.incl>

define delay tauA
define delay tauB

;DELAYS
"d0=0.0"
"in0=inf1/2"
"tauA=in0/2-p16-d16-50u"
"tauB=dw*2*cnst4"
"d11=30m"
"d16=1m"
"d17=200u"

;PULSES
"p2=p1*2"
"p4=p3*2"
"p16=1m"
```

```
"p17=1m"
"p10=p40"

;%%%%%%%%%% WaveMaker Calculations %%%%%%%%%%
;sp15:wvm:X1_wurst-20:f2 wurst-20(cnst5 kHz, cnst3 ms; Q=cnst7, L2H)
;sp19:wvm:X2_wurst-20:f2 wurst-20(cnst5 kHz, cnst3 ms; Q=cnst7, H2L)
;sp31(p63,pl12):wvm:HXdec:f2 wurst-80(cnst5 kHz, cnst10 ms; Q=cnst8, L2H) ss=1.0 us;
;
;Constants definition
"d11=30m+1s/(cnst1)-1s/(cnst1)"
"d11=30m+1s/(cnst3)-1s/(cnst3)"
"d11=30m+1s/(cnst5)-1s/(cnst5)"
"d11=30m+1s/(cnst7)-1s/(cnst7)"
"d11=30m+1s/(cnst8)-1s/(cnst8)"
"d11=30m+1s/(cnst9)-1s/(cnst9)"
"d11=30m+1s/(cnst10)-1s/(cnst10)"
"cnst9=200/cnst1"
"p63=cnst10*1000"
"plw12=spw31"

; BAND-SELECTIVE PULSE
#ifdef BS
;sp12:wvm:H_rsnob:f1 rsnob(cnst12 Hz, cnst11 ppm; PA=0.5; NPOINTS=1000) ss=1.0us;
"spoff12=0"
"d11=30m+1s/(cnst11)-1s/(cnst11)"
"d11=30m+1s/(cnst12)-1s/(cnst12)"
"d11=30m"
#   endif

; ZANGGER-STERK PULSE
#ifdef ZS
;sp12:wvm:H_rsnob:f1 rsnob(cnst12 Hz, cnst11 ppm; PA=0.5; NPOINTS=1000) ss=1.0us;
"spoff12=0"
"d11=30m+1s/(cnst11)-1s/(cnst11)"
"d11=30m+1s/(cnst12)-1s/(cnst12)"
"d11=30m"
#   endif

;SALTIRE PULSE
#   ifdef dSALTIRE
;Double Saltire pulse calculations
"cnst50=(cnst20/360)*sqrt((2*cnst21)/(p40/2000000))"
"p30=1000000.0/(cnst50*4)"
"cnst31= (p30/p1) * (p30/p1)"
"spw40=plw1/cnst31"
"spoff40=0"
#   endif

;ACQUISITION
"acqt0=0"
baseopt_echo

1 ze
  30m pl12:f2
2 30m do:f2
  50u BLKGRAMP
  50u LOCKH_OFF
  d1
  50u LOCKH_ON
  50u UNBLKGRAMP pl1:f1 pl2:f2

3

if "d0==0.0" ;chunk 1
{
p1 ph1
}
else
{
```

```
 "DELTA=d0/2-(p15/2)"
  p1 ph1
  DELTA pl0:f2
  (p15:sp15 ph4):f2
  DELTA
}
  tauA
  50u
  p16:gp1
  d16
  p2 ph2
  p16:gp1
  d16
  50u
  tauA

  tauB
  50u
  d17 pl0:f1
  p16:gp2
  d16
# ifdef BS
  10u
  (p12:sp12 ph3):f1
  10u
# endif
# ifdef ZS
  10u gron0
  (p12:sp12 ph3):f1
  10u groff
# endif
# ifdef dSALTIRE
  10u
 (center (p40:sp40 ph3):f1 (p10:gp10) )
  10u
# endif
  d17
  p16:gp2
  d16
  50u BLKGRAMP pl12:f2

if "d0==0.0"
{
}
else
{
  DELTA pl0:f2
  (p19:sp19 ph5):f2
  DELTA pl12:f2
}
  go=2 ph31 cpd2:f2
  30m do:f2 mc #0 to 2 F1QF(id0)
exit
  50u LOCKH_OFF

ph1= 0 0 0 0 1 1 1 1
ph2= 0 0 0 0 0 0 0 0 2 2 2 2 2 2 2 2
ph3= 0 1 2 3
ph4= 0
ph5= 0
ph31=0 2 0 2 1 3 1 3

;POWER LEVEL
;pl0  : f1 channel - zero power (0W)
;pl1  : f1 channel - power level for hard pulse (default)
;pl2  : f2 channel - power level for hard pulse (default)
;pl12 : f2 channel - power level for adiabatic X decoupling sequence
;sp12 : f1 channel - power level for selective H 180 refocussing pulse (BS and ZS approach)
;sp15 : f2 channel - power level for the first adiabatic X 180 inversion pulse
```

```
;sp19 : f2 channel - power level for the second adiabatic X 180 inversion pulse
;sp40 : f1 channel - power level for double-saltire pulse (PSYCHE approach)

;PULSES
;p1  : f1 channel - 90 degree high power pulse
;p2  : f1 channel - 180 degree high power pulse
;p3  : f2 channel - 90 degree high power pulse
;p4  : f2 channel - 180 degree high power pulse
;p10 : f1 channel - duration of weak gradient during double-saltire element (PSYCHE approach)
;p12 : f1 channel - duration of 180 H selective refocussing pulse (BS and ZS approach)
;p15 : f2 channel - duration of the first adiabatic pulse in X channel
;p16 : f1 channel - duration of CTP gradients of hard H 180 pulse        [1 ms]
;p17 : f1 channel - duration of CTP gradients of selective H 180 pulse  [1 ms]
;p19 : f2 channel - duration of the second adiabatic pulse in X channel
;p40 : f1 channel - duration of double-saltire element (PSYCHE approach)
;p63 : f2 channel – duration of the adiabatic pulse in CPD

;DELAYS
;d0  : incremented delay
;d1  : relaxation delay;  > 2 T1
;d16 : recovery delay for CTP gradients of hard 180 pulse       [1 ms]
;d17 : recovery delay                                            [200 us]
;tauA: delay according to chunk duration
;tauB: digital filter correction delay

;SHAPED PULSES
;spnam12: f1 channel - file name for the 1H 180 selective refocussing pulse (BS and ZS approach)     [RSNOB]
;spnam15: f2 channel - file name for the first adiabatic X inversion pulse    [WURST-20]
;spnam19: f2 channel - file name for the second adiabatic X inversion pulse  [WURST-20]
;spnam40: f1 channel - file name for the double-saltire pulse element (PSYCHE approach)   [SALTIRE]

;GRADIENTS
;gpz0: spatial encoding gradient during 1H 180 selective refocussing pulse (ZS approach)     [0.1-2%]
;gpz1: CTP gradient for hard 180 pulse      [79%]
;gpz2: CTP gradient for selective 180 pulse [47%]
;gpz10: weak gradient during double-saltire pulse element (PSYCHE approach)   [1-3%]
;gpnam1: SINE.100
;gpnam2: SINE.100
;gpnam10: RECT.1

;CONSTANTS
;cnst1 : largest J(HX) value [Hz]
;cnst3 : f2 channel - duration of adiabatic X inversion pulses [< 5 ms]
;cnst4 : number of drop points                       [4]
;cnst5 : f2 channel - bandwidth for adiabatic X pulses and decoupling sequence [kHz]
;cnst7 : f2 channel - Q factor for adiabatic 180 X inversion pulses [<5]
;cnst8 : f2 channel - Q factor for adiabatic X decoupling sequence [typically 2-3]
;cnst9 : f2 channel - recommended X decoupling sequence duration
;cnst10: Set as cnst9 (or <5-10 ms, depending on chunk duration)
;cnst11: f1 channel - chemical shift for BS selective pulse. If ZS: set as o1p (in ppm)
;cnst12: f1 channel - bandwidth for selective refocussing pulse (in Hz)
;cnst20: f1 channel - desired flip angle for double-saltire pulse element [10-25 degrees]
;cnst21: f1 channel - bandwidth of each saltire pulse [10000 Hz}

;OTHER
;TD1 : number of t1 increments        [16-64]
;in0 : 1/(2 * SW) = DW
;nd0 : 2
;IN_F: chunk duration [us]
;MC2 : QF
;ns : 2 * n
;ds : 2
;BS - set cnst11 to peak of interest
;ZS and PSYCHE: set O1 on in the centre of the spectral window
;cpd2: p5m4sp180.2 (use wvm -a command for power parameters)
```

b. ‘Small J and δ’ pulse program suitable for a TBI probe

```
;X2_dec_if_id_ts4x_tbi
;Alternative pulse sequence for X decoupled pure shift 1D experiment with interferogram acquisition
;Recommended only for small JHX values and a small heteronuclear chemical shift range
;
;Developed by:
;NMR Methodology Group
;University of Manchester
;
;Pure shift 1D experiment
;     with BB heteronuclear and homonuclear decoupling
;     with interferogram acquisition
;
;Data can be reconstructed using the “pshift4f” macro available at
https://www.nmr.chemistry.manchester.ac.uk/sites/default/files/pshift4f
;
;References:
;Heteronuclear decoupled: C. Mycroft, M. Nilsson, G. A. Morris, L. Castanar, Simultaneous broadband
suppression of homonuclear and heteronuclear couplings in 1H NMR spectroscopy (2022)
;Interferogram acquisition:  K. Zangger, H. Sterk, J. Magn. Reson., 124, 486-489 (1997)
;                            J. A. Aguilar, S. Faulkner, M. Nilsson, G. A. Morris, 49, 3901-3903 (2010)
;Band-selective: L. Castanar, P. Nolis, A. Virgili, T. Parella, Chem. Eur. J., 19, 17283-17286 (2013)
;                J. Ying, J. Roche, A. Bax, J. Magn. Reson., 241, 97-102 (2014)
;Zangger-Sterk:  K. Zangger, H. Sterk, J. Magn. Reson., 124, 486-489 (1997)
;PSYCHE: M. Foroozandeh, R. W. Admas, N. J. Meharry, D. Jeannerat, M. Nilsson, G. A. Morris, Angew. Chem.
Int. Ed., 53, 6990-6992 (2014)
;
;THE USE OF WAVEMAKER IS RECOMMENDED
;
;Pure shift active spin refocusing options
;      ZGOPTION= -DBS (Band-Selective)
;      ZGOPTION= -DZS (Zangger-Sterk)
;      ZGOPTION= -DdSALTIRE (PSYCHE) [used by default]
;
;Avance NEO Version
;Topspin.4x
;
;$CLASS=HighRes
;$DIM=2D
;$TYPE=
;$SUBTYPE=
;$COMMENT=

#include <Avance.incl>
#include <Delay.incl>
#include <Grad.incl>

define delay tauA
define delay tauB

;%%%%%%%%%% WaveMaker Calculations %%%%%%%%%%
; BAND-SELECTIVE PULSE
#ifdef BS
;sp12:wvm:H_rsnob:f1 rsnob(cnst12 Hz, cnst11 ppm; PA=0.5; NPOINTS=1000) ss=1.0 us;
"spoff12=0"
"d11=30m+1s/(cnst11)-1s/(cnst11)"
"d11=30m+1s/(cnst12)-1s/(cnst12)"
"d11=30m"
#   endif
```

```
; ZANGGER-STERK PULSE
#ifdef ZS
;sp12:wvm:H_rsnob:f1 rsnob(cnst12 Hz, cnst11 ppm; PA=0.5; NPOINTS=1000) ss=1.0 us;
"spoff12=0"
"d11=30m+1s/(cnst11)-1s/(cnst11)"
"d11=30m+1s/(cnst12)-1s/(cnst12)"
"d11=30m"
#   endif

;SALTIRE PULSE
#   ifdef dSALTIRE
;Double Saltire pulse calculations
"cnst50=(cnst20/360)*sqrt((2*cnst21)/(p40/2000000))"
"p30=1000000.0/(cnst50*4)"
"cnst31= (p30/p1) * (p30/p1)"
"spw40=plw1/cnst31"
"spoff40=0"
#   endif

;DELAYS
"d0=0"
"in0=inf1/2"
"tauA=in0/2-p16-d16-50u"
"tauB=dw*2*cnst4"
"d11=30m"
"d16=1m"
"d17=200u"

;PULSES
"p2=p1*2"
"p4=p3*2"
"p16=1m"
"p17=1m"
"p10=p40"

;ACQUISITION
"acqt0=0"
baseopt_echo

1 ze
2 d11
  50u BLKGRAMP
  50u LOCKH_OFF
  d1 pl1:f1 pl2:f2
  50u LOCKH_ON
  50u UNBLKGRAMP

3 p1 ph1
  d0
  tauA
  50u
  p16:gp1
  d16
 (center (p2 ph2):f1 (p4 ph4):f2 )
  p16:gp1
  d16
  50u
  tauA

  tauB
```

```
  50u
  d17 pl0:f1
  p17:gp2
  d16
# ifdef BS
  10u
  (p12:sp12 ph3):f1
  10u
# endif
# ifdef ZS
  10u gron0
  (p12:sp12 ph3):f1
  10u groff
# endif
# ifdef dSALTIRE
  10u
 (center (p40:sp40 ph3):f1 (p10:gp10) )
  10u
# endif
  d17
  p17:gp2
  d16
  50u BLKGRAMP
  d0

  go=2 ph31
  d11 mc #0 to 2 F1QF(id0)
exit
  50u LOCKH_OFF

ph1= 0 0 0 0 1 1 1 1
ph2= 0 0 0 0 0 0 0 0 2 2 2 2 2 2 2 2
ph3= 0 1 2 3
ph4= 0
ph31=0 2 0 2 1 3 1 3

;POWER LEVEL
;pl0  : f1 channel - zero power (0W)
;pl1  : f1 channel - power level for hard pulse (default)
;pl2  : f2 channel - power level for hard pulse (default)
;sp12 : f1 channel - power level for selective H 180 refocussing pulse (BS and ZS approach)
;sp40 : f1 channel - power level for double-saltire pulse (PSYCHE approach)

;PULSES
;p1  : f1 channel - 90 degree high power pulse
;p2  : f1 channel - 180 degree high power pulse
;p3  : f2 channel - 90 degree high power pulse
;p4  : f2 channel - 180 degree high power pulse
;p10 : f1 channel - duration of weak gradient during double-saltire element (PSYCHE approach)
;p12 : f1 channel - duration of 180 H selective refocussing pulse (BS and ZS approach)
;p16 : f1 channel - duration of CTP gradients of hard H 180 pulse       [1 ms]
;p17 : f1 channel - duration of CTP gradients of selective H 180 pulse  [1 ms]
;p40 : f1 channel - duration of double-saltire element (PSYCHE approach)

;DELAYS
;d0  : incremented delay
;d1  : relaxation delay;  > 2 T1
;d16 : recovery delay for CTP gradients of hard 180 pulse       [1 ms]
;d17 : recovery delay                                           [200 us]
;tauA: delay according to chunk duration
```

;tauB: digital filter correction delay

;SHAPED PULSES
;spnam12: f1 channel - file name for the 1H 180 selective refocussing pulse (BS and ZS approach) [RSNOB]
;spnam40: f1 channel - file name for the double-saltire pulse element (PSYCHE approach) [SALTIRE]

;GRADIENTS
;gpz0: spatial encoding gradient during 1H 180 selective refocussing pulse (ZS approach) [0.1-2%]
;gpz1: CTP gradient for hard 180 pulse [79%]
;gpz2: CTP gradient for selective 180 pulse [47%]
;gpz10: weak gradient during double-saltire pulse element (PSYCHE approach) [1-3%]
;gpnam1: SINE.100
;gpnam2: SINE.100
;gpnam10: RECT.1

;CONSTANTS
;cnst4: number of drop points [4]
;cnst11: f1 channel - chemical shift for BS selective pulse. If ZS: set as o1p (in ppm)
;cnst12: f1 channel - bandwidth for selective refocussing pulse (in Hz)
;cnst20: f1 channel - desired flip angle for double-saltire pulse element [10-25 degrees]
;cnst21: f1 channel - bandwidth of each saltire pulse [10000 Hz}

;OTHER
;TD1 : number of t1 increments [16-64]
;in0 : 1/(2 * SW) = DW
;nd0 : 2
;IN_F: chunk duration [us]
;MC2 : QF
;ns : 2 * n
;ds : 2
;BS - set cnst11 to peak of interest
;ZS and PSYCHE: set O1 on in the centre of the spectral window

7. References

1 L. Castañar, P. Nolis, A. Virgili and T. Parella, *Chem. Eur. J.*, 2013, **19**, 17283–17286.
2 R. W. Adams, L. Byrne, P. Király, M. Foroozandeh, L. Paudel, M. Nilsson, J. Clayden and G. A. Morris, *Chem. Commun.*, 2014, **50**, 2512–2514.
3 J. Ying, J. Roche and A. Bax, *J. Magn. Reson.*, 2014, **241**, 97–102.
4 K. Zangger and H. Sterk, *J. Magn. Reson.*, 1997, **489**, 486–489.
5 M. Foroozandeh, R. W. Adams, N. J. Meharry, D. Jeannerat, M. Nilsson and G. A. Morris, *Angew. Chem. Int. Ed.*, 2014, **53**, 6990–6992.
6 M. Foroozandeh, G. A. Morris and M. Nilsson, *Chem. Eur. J.*, 2018, **24**, 13988–14000.
7 J. A. Aguilar, S. Faulkner, M. Nilsson and G. A. Morris, *Angew. Chem. Int. Ed.*, 2010, **49**, 3901–3903.
8 A. J. Shaka, J. Keeler, T. A. Frenkiel and R. Freeman, *J. Magn. Reson.*, 1983, **52**, 335–338.
